# Edge-Passivated Monolayer WSe_2_ Nanoribbon Transistors

**DOI:** 10.1002/adma.202313694

**Published:** 2024-07-18

**Authors:** Sihan Chen, Yue Zhang, William P. King, Rashid Bashir, Arend M. van der Zande

**Affiliations:** Holonyak Micro and Nanotechnology Laboratory, University of Illinois Urbana-Champaign, Urbana, IL 61801, USA; Department of Mechanical Science and Engineering, University of Illinois Urbana-Champaign, Urbana, IL 61801, USA; Holonyak Micro and Nanotechnology Laboratory, University of Illinois Urbana-Champaign, Urbana, IL 61801, USA; Department of Mechanical Science and Engineering, University of Illinois Urbana-Champaign, Urbana, IL 61801, USA; Materials Research Laboratory, University of Illinois Urbana-Champaign, Urbana, IL 61801, USA; Holonyak Micro and Nanotechnology Laboratory, University of Illinois Urbana-Champaign, Urbana, IL 61801, USA; Department of Bioengineering, University of Illinois Urbana-Champaign, Urbana, IL 61801, USA; Holonyak Micro and Nanotechnology Laboratory, University of Illinois Urbana-Champaign, Urbana, IL 61801, USA; Department of Mechanical Science and Engineering, University of Illinois Urbana-Champaign, Urbana, IL 61801, USA; Materials Research Laboratory, University of Illinois Urbana-Champaign, Urbana, IL 61801, USA

**Keywords:** edge passivation, monolayer, nanoribbon, scanning probe lithography, transistors, tungsten oxyselenide, WSe2

## Abstract

The ongoing reduction in transistor sizes drives advancements in information technology. However, as transistors shrink to the nanometer scale, surface and edge states begin to constrain their performance. 2D semiconductors like transition metal dichalcogenides (TMDs) have dangling-bond-free surfaces, hence achieving minimal surface states. Nonetheless, edge state disorder still limits the performance of width-scaled 2D transistors. This work demonstrates a facile edge passivation method to enhance the electrical properties of monolayer WSe_2_ nanoribbons, by combining scanning transmission electron microscopy, optical spectroscopy, and field-effect transistor (FET) transport measurements. Monolayer WSe_2_ nanoribbons are passivated with amorphous WO_x_Se_y_ at the edges, which is achieved using nanolithography and a controlled remote O_2_ plasma process. The same nanoribbons, with and without edge passivation are sequentially fabricated and measured. The passivated-edge nanoribbon FETs exhibit 10 ± 6 times higher field-effect mobility than the open-edge nanoribbon FETs, which are characterized with dangling bonds at the edges. WO_x_Se_y_ edge passivation minimizes edge disorder and enhances the material quality of WSe_2_ nanoribbons. Owing to its simplicity and effectiveness, oxidation-based edge passivation could become a turnkey manufacturing solution for TMD nanoribbons in beyond-silicon electronics and optoelectronics.

## Graphical Abstract

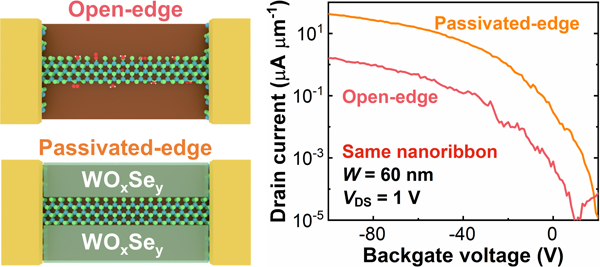

This study presents edge passivation for p-type monolayer WSe_2_ nanoribbon transistors using nanolithography and a controlled remote O_2_ plasma process. The edge passivation material consists of amorphous WO_x_Se_y_. Compared to microribbons, nanoribbons with passivated edges exhibit reduced defect density and increased p-doping, whereas nanoribbons with open edges exhibit higher defect densities and reduced p-doping.

## 1. Introduction

The continuous downscaling of transistors has been a major driving force behind the advancement of very-large-scale-integration (VLSI) technologies by offering better performance, higher integration density, and lower power consumption.^[1]^ However, as the physical dimensions of the transistors approach sub-10 nm, the effects of surface and edge states of the semiconducting channel become pronounced, limiting the transistor performance.^[2,3]^ These electronic states introduce disorder and charge traps, and decrease the carrier mobility.^[4,5]^ For instance, the carrier mobility of silicon significantly drops as its channel thickness decreases into the sub-5 nm regime due to charge scattering at the interfaces.^[4]^

On the other hand, 2D semiconductors like transition metal dichalcogenides (TMDs) such as MoS_2_ and WSe_2_, have dangling-bond-free surfaces and hence achieve minimal surface states.^[6]^ Consequently, their carrier mobility is not strongly affected by surface scattering and can remain high even at the atomic thickness limit.^[7]^ As a result, 2D semiconductors are being considered for beyond-silicon complementary metal–oxide–semiconductor (CMOS) technologies. However, when 2D materials are patterned into nanoribbons, the edge disorder begins to dominate, leading to drastic reductions in performance. Therefore, a key line of research is in how to engineer and passivate the edges of nanoribbons to maintain the performance of microribbons.^[8]^

TMD nanoribbon field-effect transistors (FETs) are fabricated using either top-down or bottom-up approaches. Top-down fabrication typically starts with micron-sized or larger TMD flakes, which are then shaped into nanoribbons using nanolithography and etching. Nanolithography techniques such as electron beam lithography (EBL),^[9–11]^ scanning probe lithography (SPL),^[12]^ and nanowire lithography^[13]^ have been used to create TMD nanoribbon FETs as narrow as 30–65 nm. Bottom-up synthesis of integration-ready TMD nanoribbons has been realized using lateral control of crystal growth^[14,15]^ or nanowire templates,^[16]^ producing nanoribbons with widths as narrow as 8–65 nm. However, FETs based on bottom-up synthesized TMD nanoribbons have yet to overcome the disorder from edge states, which limits their performance compared to top-down approaches.^[14–17]^ The best monolayer TMD nanoribbon n-FET with a 50 nm channel width has a field-effect mobility of 50 cm^2^ V^−1^ s^−1^,^[10]^ while the best TMD nanoribbon p-FET, with a 50 nm channel width and 4.9 nm thickness, has a mobility of 3 cm^2^ V^−1^ s^−1^.^[17]^ In comparison, monolayer MoS_2_ microribbon n-FETs have achieved a mobility of 167 cm^2^ V^−1^ s^−1^,^[18]^ and bilayer WSe_2_ microribbon p-FETs have reached 137 cm^2^ V^−1^ s^−1^.^[19]^ The significant performance gap between TMD microribbon and nanoribbon FETs, particularly within p-FETs, suggests that edge states significantly constrain the electrical performance of TMD nanoribbons.

To fabricate high-performance TMD nanoribbon transistors, minimizing edge states is essential. One approach is to create atomically smooth edges.^[20]^ Although bottom-up synthesis has produced monolayer MoS_2_ and MoSe_2_ nanoribbons with smooth edges, these structures have yet to be integrated into working transistors.^[21–23]^ Another effective method is edge passivation, which eliminates dangling bonds and stabilizes edge atoms.^[24,25]^ This approach significantly reduces edge disorder and scattering, thereby enhancing the electrical performance and stability of nanoribbons.^[24,26,27]^ For instance, ab initio simulations show that dangling bonds introduce in-gap states for un-passivated graphene nanoribbons (GNRs), a phenomenon absent in hydrogen-passivated GNRs.^[24]^ Similarly, scanning tunneling spectroscopy (STS) revealed that air-oxidized WSe_2_ edges have a band gap 1.08 eV larger than WSe_2_ terraces, resulting in the electronic passivation of WSe_2_.^[27]^ Edge passivation is also compatible with large-scale, top-down fabrication. Thus, a simple and CMOS-compatible edge passivation method is crucial for manufacturing high-performance, ultra-scaled TMD transistors.^[28]^

In this article, we report a facile edge passivation method for monolayer WSe_2_ nanoribbon p-FETs, achieving up to two orders of magnitude enhancement in the on-state current. We fabricate these edge-passivated nanoribbons using nanolithography and a controlled remote O_2_ plasma process. Scanning transmission electron microscopy (STEM) identifies the passivation material as amorphous WO_x_Se_y_. Photoluminescence (PL) and transport studies show that WO_x_Se_y_ edge passivation significantly reduces edge disorder. The electrical properties of microribbons are preserved and even improved in edge-passivated nanoribbons, offering a viable method to integrate TMD monolayers into nanoribbon FETs without compromising performance. Monolayer WSe_2_ nanoribbon p-FETs with WO_x_Se_y_-passivated edges effectively bridge the performance gap between TMD nanoribbon n-FETs and p-FETs.

We choose WO_x_Se_y_ for edge passivation for four reasons: First, density functional theory calculations reveal that oxygen passivation eliminates the in-gap states introduced by selenium vacancies in WSe_2_.^[29–31]^ First-principle transport calculations demon- strate that filling chalcogen vacancies with oxygen atoms en- hances the carrier mobility of TMDs (e.g., WSe_2_, MoS_2_, MoSe_2_, WS_2_) due to an increased Drude relaxation time.^[32]^ Second, WO_x_Se_y_ can be readily formed with atomic layer precision by oxidizing the outmost WSe_2_ layer via controlled O_2_ plasma^[33]^ or UV/ozone,^[34]^ which is desired for heterogeneous integration of 2D materials.^[35]^ Third, WO_x_Se_y_ is stable under ambient conditions, making it compatible with transistor fabrication.^[33,36]^ Fourth, WO_x_Se_y_ is a p-type surface charge transfer dopant.^[36]^ In-plane charge transfer from edge-bound WO_x_Se_y_ may induce hole doping in WSe_2_ nanoribbons.

## 2. Results and Discussion

### 2.1. Fabrication of Monolayer WSe_2_ Nanoribbons

**Figure 1.**
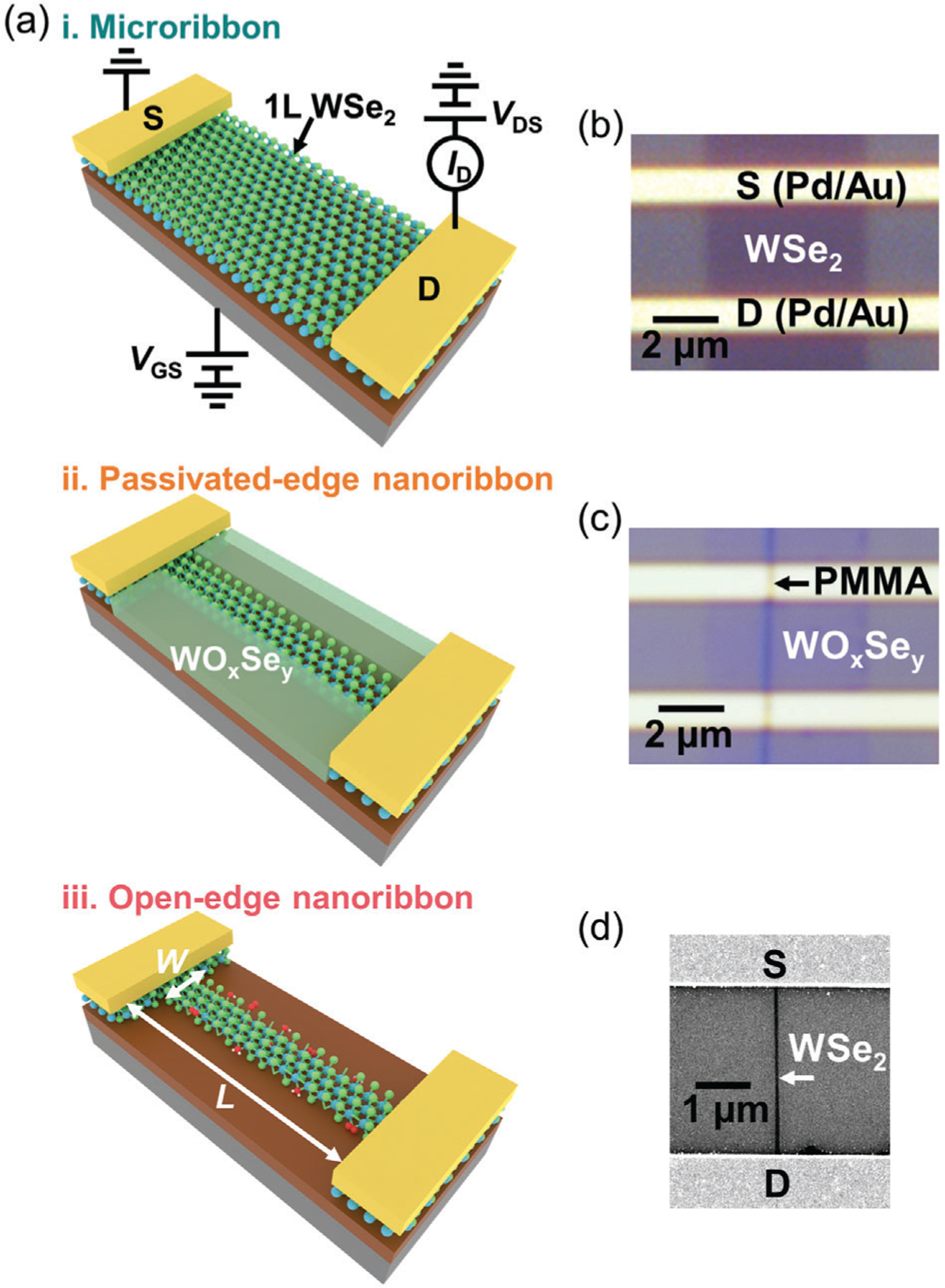
a) Schematics of the same monolayer WSe_2_ FET through sequential fabrication steps as a i) microribbon, as a ii) passivated-edge nanoribbon, and as an iii) open-edge nanoribbon. All WSe_2_ nanoribbons have a PMMA nanoribbon mask on top, which is not shown in the schematics for clarity. b) Optical image of a monolayer WSe_2_ microribbon FET. c) Optical image of a monolayer WSe_2_ nanoribbon FET with WO_x_Se_y_ passivated edges with a PMMA nanoribbon mask on top. d) SEM image of a monolayer WSe_2_ nanoribbon FET with open edges (Device B in Table S3, Supporting Information). All the SEM images in this article were taken after the removal of PMMA using solvents.

To investigate the effects of WO_x_Se_y_ edge passivation on the optical and electrical properties of WSe_2_ nanoribbons, we sequentially fabricated and measured the same nanoribbons with different edge structures – passivated and open edges. The passivated-edge nanoribbon refers to the nanoribbon with edge atoms covalently bonded to WO_x_Se_y_. The open-edge nanoribbon refers to the nanoribbon with dangling bonds and defects at the edges. Figure 1 shows the fabrication process of monolayer WSe_2_ nanoribbon FETs. As illustrated in Figure 1a, we fabricated and measured the same monolayer WSe_2_ FETs with three different channel structures – as a i) microribbon, as a ii) passivated-edge nanoribbon, and as an iii) open-edge nanoribbon. Each FET consists of a monolayer WSe_2_ channel, electrical contacts for the source (S) and drain (D) consisting of 50 nm gold on 5 nm palladium, a 285 nm SiO_2_ gate dielectric, and a degenerately p-doped silicon back gate. Figure 1b shows an optical image of the starting monolayer WSe_2_ microribbon FET. Raman and PL spectra in Figure S1, Supporting Information, confirm the monolayer structure. Figure 1c shows an optical image of the same FET after patterning the channel into a nanoribbon with passivated edges. Figure 1d shows the scanning electron microscopy (SEM) image of the same FET after the removal of WO_x_Se_y_, resulting in an open-edge nanoribbon FET.

The detailed fabrication process is depicted in Figure S2, Supporting Information, and Experimental Section provide all process parameters. Briefly, we use thermal dip-pen nanolithography (tDPN) to deposit a PMMA nanoribbon mask onto the microribbon FET,^[12,37]^ and then expose the FET to a remote O_2_ plasma with 50 watts power for 20 s to oxidize the un-masked WSe_2_. As we will show, the low-energy remote O_2_ plasma chemically modifies the WSe_2_ rather than etching it, producing amorphous WO_x_Se_y_, which bonds to and passivates the edges of the nanoribbon. Finally, We etch the WO_x_Se_y_ by dipping the sample in 1 M KOH for 10 s.^[34]^ KOH selectively removes WO_x_Se_y_ without etching WSe_2_ or Pd/Au.^[34,38]^ In addition, the PMMA mask is left on the nanoribbon throughout the whole process to minimize changes in dielectric environment and doping during the processing steps. As a result of removing the edge passivation, the open-edge nanoribbon will have uncontrolled dangling bonds and defects at the edges. For example, ambient molecules such as H_2_O and O_2_ tend to adsorb onto the open edges.^[39]^ The WO_x_Se_y_ results from the self-limiting oxidation of the topmost layer of WSe_2_.^[33,34]^ Thus, by its nature, WO_x_Se_y_ edge passivation can only work with monolayer WSe_2_.

In sub-sections 2.2 and 2.3, we will show nanoribbons integrated onto TEM grids for atomic structural characterization and nanoribbon arrays for optical characterization. The differences in preparing these samples are illustrated in Figures S3 and S4, Supporting Information, but are conceptually the same as the FET fabrication process.

### 2.2. STEM of Monolayer WSe_2_ with WO_x_Se_y_ Passivated Edges and Open Edges

**Figure 2.**
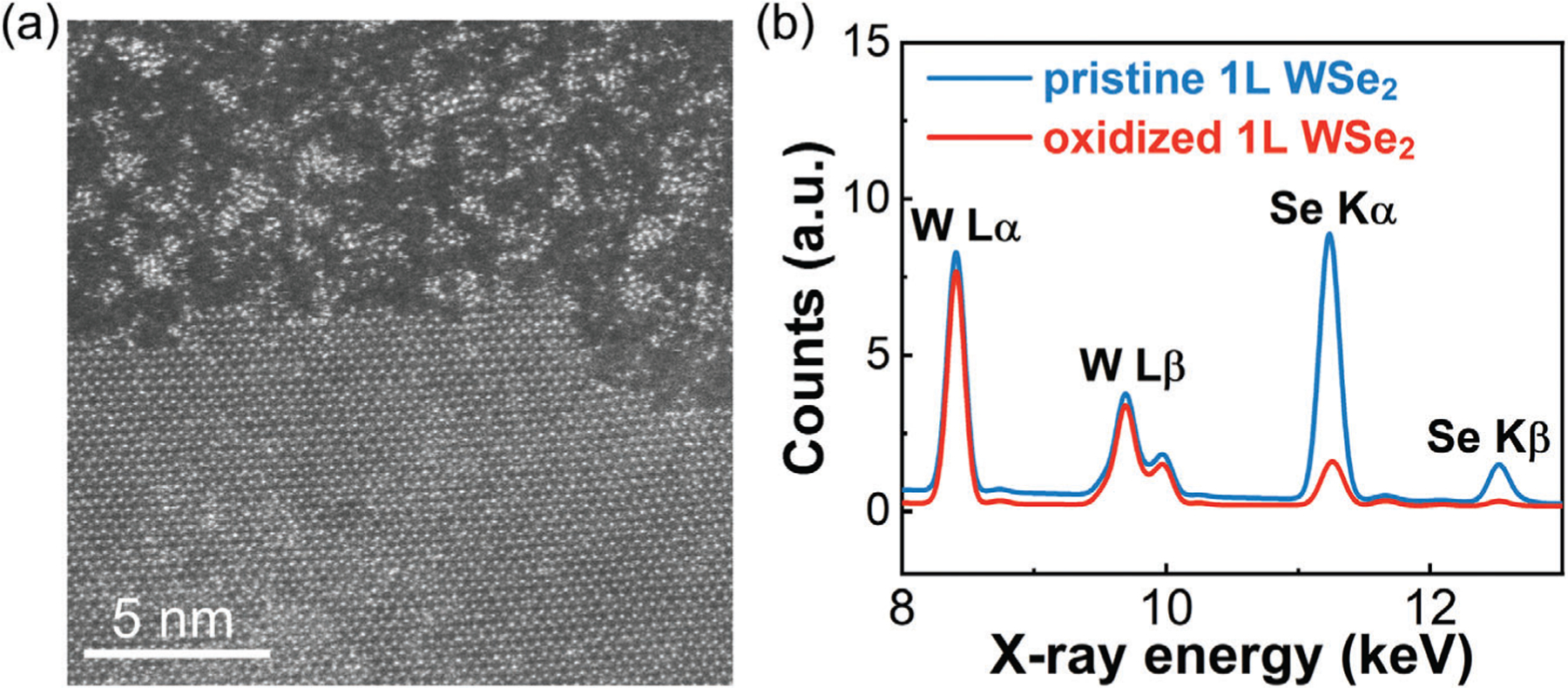
a) HAADF-STEM image of monolayer WSe_2_ with an oxidized edge. b) Area-integrated EDS spectra of both pristine and oxidized monolayer (1L) WSe_2_ regions.

When applying monolayer TMD nanoribbon transistors at advanced VLSI nodes, the width of the nanoribbons could eventually be as small as 5–10 nm,^[8]^ so the exact structure of the edges will play a critical role on the transistor performance. Before we investigate the properties of monolayer WSe_2_ nanoribbons, we first analyze the atomic structure and chemistry of the oxidized edges of monolayer WSe_2_ using high-angle annular dark-field (HAADF) STEM and energy dispersive X-ray spectroscopy (EDS) in Figure 2. Figure 2a shows the STEM image of monolayer WSe_2_ with an oxidized edge after annealing at 200 °C in vacuum. The oxidized WSe_2_ appeared amorphous yet seamlessly connected to the crystalline monolayer WSe_2_ at the edge. Tungsten atoms formed clusters within the oxidized WSe_2_. Figure 2b plots the integrated EDS spectra of pristine WSe_2_ and oxidized WSe_2_ regions (see Figure S5, Supporting Information for the EDS map). The oxidized WSe_2_ exhibited W peaks nearly identical to the pristine WSe_2_, but significantly smaller yet discernible Se peaks. Thus, we hypothesize that the oxidized WSe_2_ constitutes WO_x_Se_y_, rather than being purely WO_x_. Previous studies have also observed the formation of WO_x_Se_y_ by oxidizing WSe_2_ using a controlled O_2_ plasma or UV/ozone process.^[33,36,40,41]^ We compare the relative Se K𝛼 peak intensities between WO_x_Se_y_ and WSe_2_ and derive the atomic selenium ratio y to be 0.4.

We attribute the aggregation of W atoms observed in WO_x_Se_y_ in Figure 2a to the combined effects of pre-STEM imaging annealing and electron beam irradiation. Annealing was necessary to minimize carbon deposition during atomic resolution STEM imaging. Figure S6, Supporting Information demonstrates that annealing at 200 °C leads to increased inhomogeneity in amorphous monolayer WO_x_Se_y_ compared to annealing at 120 °C. Furthermore, Figure S7, Supporting Information demonstrates the continuous aggregation of W atoms in amorphous monolayer WO_x_Se_y_ under successive electron beam irradiation. Since the passivated-edge nanoribbon FETs were fabricated and measured without undergoing annealing or irradiation, we infer that the oxidized edges of these nanoribbons were more homogeneous than depicted in Figure 2a.

Surprisingly, the amorphous WO_x_Se_y_ was stable and free-standing as a monolayer, which is remarkable given the historical challenge of obtaining stable amorphous 2D monolayers.[42,43] The observation that the amorphous monolayer WO_x_Se_y_ maintained its structural integrity as a continuous film, allowing the movement of W atoms to form clusters, indicates the possibility of Se_y_O_x_ to independently form a stable amorphous monolayer. For context, the crystal structure of SeO_2_ consists of flat chains, which could theoretically create 2D networks.^[44]^ In addition, the Se_y_O_x_ within WO_x_Se_y_ undergoes reduction at 200 °C,^[41]^ suggesting that the Se peaks of WO_x_Se_y_ in Figure 2b and the value of y would have been larger without annealing.

Figure S8, Supporting Information shows STEM images of etched edges following the removal of WO_x_Se_y_ with a KOH bath. As expected, the etched edges were devoid of passivating solid-state materials and hence called open edges. These open edges, mostly in a zigzag pattern, were predominantly terminated with W atoms, implying a lower concentration of p-doping near the edges compared to the material’s interior.^[45]^

### 2.3. Optical Properties of Monolayer WSe2 Nanoribbons

**Figure 3.**
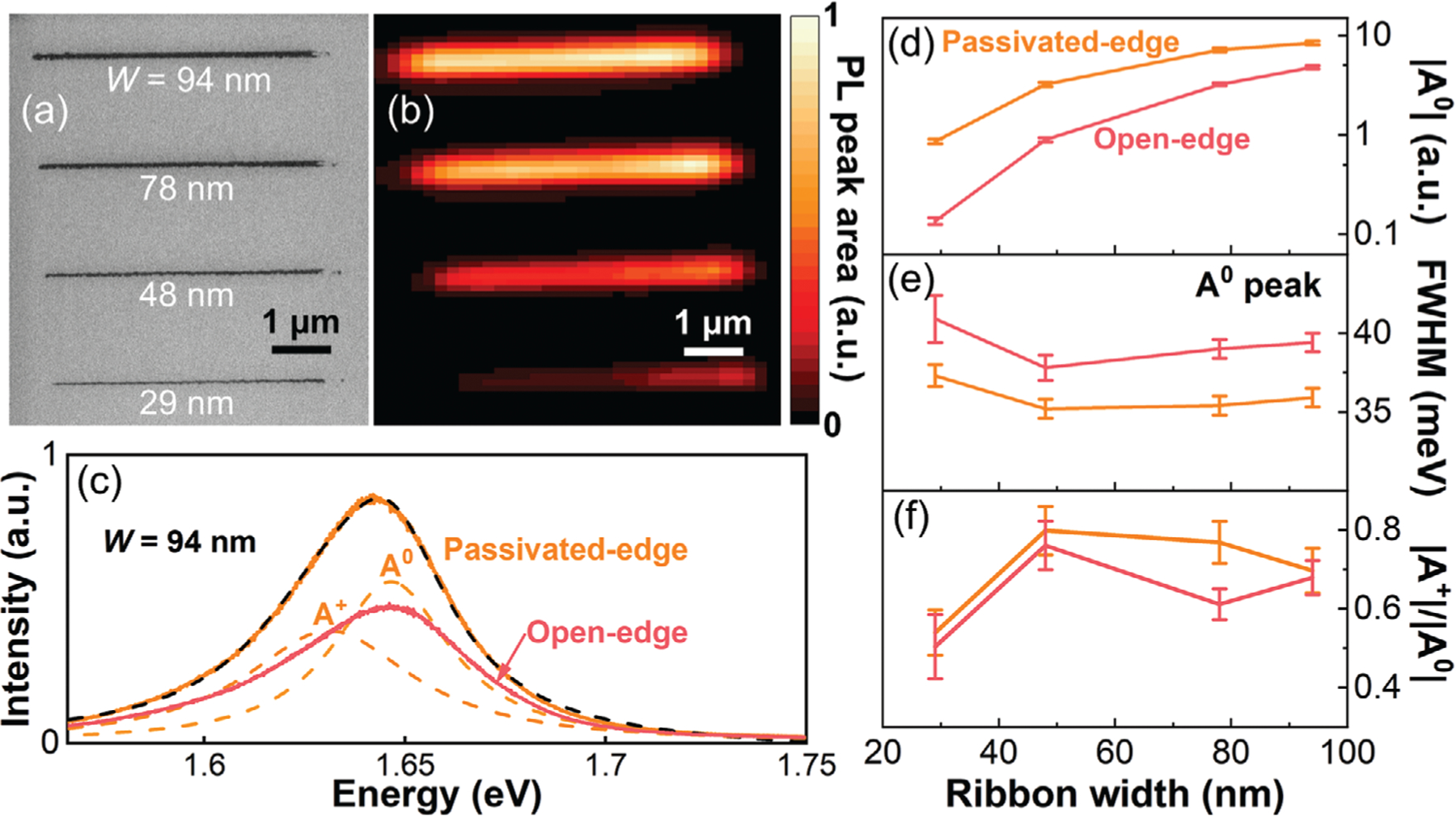
PL spectroscopy of passivated-edge and open-edge monolayer WSe_2_ nanoribbons. a) SEM image of the nanoribbons used in the PL measurements. b) PL peak area map of passivated-edge nanoribbons. c) PL spectra of a 94-nm-wide nanoribbon with both edge structures. The PL spectrum of the passivated-edge nanoribbon is fitted as a sum (dashed black line) of two Lorentzian curves (dashed orange lines) representing the exciton state (A^0^) and the trion state (A^+^). Nanoribbon width dependence of d) the exciton peak intensity |A^0^| in log scale, e) the exciton peak linewidth (FWHM), and f) the relative peak intensity |A^+^|/|A^0^|, for both edge structures.

In Figure 3, we compare the PL characteristics of an array of monolayer WSe_2_ nanoribbons with different edge structures using PL spectroscopy mapping, which gives a measure of the material quality through the nanoribbon edge passivation process. Figure S9, Supporting Information provides additional Raman characterization, which confirms the preservation of the crystal structure of monolayer WSe_2_ nanoribbons after KOH etch. Figure 3a is an SEM image of an array of nanoribbons with width ranging from 29 to 94 nm. Figure 3b is a map of the integrated PL intensity of the nanoribbon array with WO_x_Se_y_ edge passivation. Figure S10, Supporting Information provides the corresponding map for the open-edge nanoribbons. Figure 3c shows the PL spectra of the 94-nm-wide nanoribbon with both passivated (orange) and open (pink) edges. We enhanced the signal-to-noise ratio by averaging the data obtained from the center of the nanoribbons in the PL maps. Figure S11a–c, Supporting Information plots the individual PL spectra of the other nanoribbons. We fitted each PL spectrum using two Lorentzian curves to correspond to the neutral exciton state (A^0^) and the trion state (A^+^). Figure 3d–f respectively plots the A^0^ intensity, the A^0^ linewidth (full width at half maximum, FWHM), and the relative peak intensity |A^+^|/|A^0^| versus the nanoribbon width.

Figure 3d shows a decrease in the intensity of A^0^ peak with ribbon width for both edge structures. This trend is unsurprising and likely corresponds with a decrease in the monolayer area for smaller nanoribbons, considering a much larger laser spot size of ≈1.0 μm. Additionally, the passivated-edge nanoribbons exhibited 76–529% larger exciton peak intensities compared to the open-edge nanoribbons, with narrower nanoribbons showing greater difference in the PL peak intensities. Figure 3e reveals that the passivated-edge nanoribbons had on average 3.3 ± 0.4 meV narrower A^0^ peak linewidth than the open-edge nanoribbons. Furthermore, Figure 3f shows that the passivated-edge nanoribbons exhibited 3–26% larger |A^+^|/|A^0^| ratio compared to the open-edge nanoribbons. Figure S11d–g, Supporting Information provides additional extracted parameters which support these findings.

The PL of TMDs are sensitive to material quality, doping, and strain.^[46–51]^ In TMD monolayers, both the PL peak intensity and linewidth reflect the level of disorder within the material,^[46–49]^ and the peak position is sensitive to doping and strain.^[50,51]^ Therefore, these PL maps provide insights into the effects of WO_x_Se_y_ edge passivation on the electrical properties of monolayer WSe_2_ nanoribbons. First, passivated-edge nanoribbons exhibited larger intensities and narrower linewidths of both A^0^ and A^+^ peaks compared to open-edge nanoribbons, suggesting a lower defect density and superior material quality of passivated-edge nanoribbons. Second, narrower nanoribbons showed a larger percentage increase in PL peak intensities due to edge passivation, implying that the improved material quality of nanoribbons with passivated edges is a result of reduced edge defects. Third, a slightly but consistently larger |A^+^|/|A^0^| ratio in passivated-edge nanoribbons suggests a light hole doping caused by WO_x_Se_y_ edge passivation. Overall, WO_x_Se_y_ edge passivation significantly reduces edge disorder and enhances the material quality of WSe_2_ nanoribbons, while lightly p-doping the nanoribbons.

### 2.4. FET Transport of Monolayer WSe2 Nanoribbons

**Figure 4.**
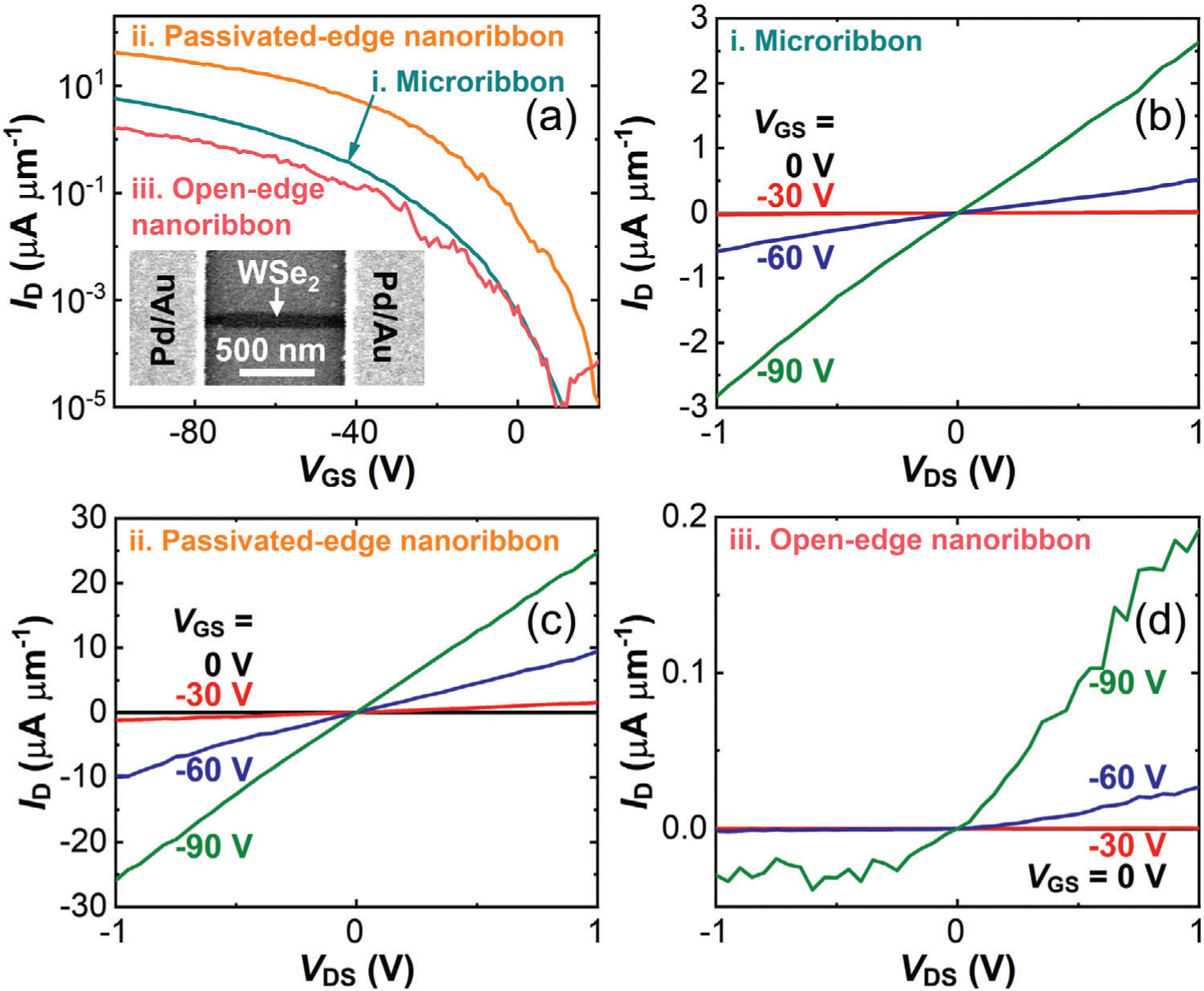
Electrical performance of a monolayer WSe_2_ FET (Device E). a) Transfer curves of the same FET with three different channel structures – as a microribbon, as a passivated-edge nanoribbon, and as an open-edge nanoribbon. *V*_DS_ = 1 V. Inset: SEM image of the nanoribbon FET with open edges. *W* = 60 nm and *L* = 970 nm. b) Output curves of the microribbon FET. c) Output curves of the nanoribbon FET with WO_x_Se_y_ passivated edges. d) Output curves of the nanoribbon FET with open edges.

We fabricated and measured seven monolayer WSe_2_ FETs (Devices A–G) sequentially through three structures: (i) microribbon (*W* = 5 μm), (ii) passivated-edge nanoribbon (*W* = 40–70 nm), and (iii) open-edge nanoribbon (*W* = 40–70 nm). Figure 4 shows the transfer (Figure 4a) and output characteristics (Figure 4b–d) of one example monolayer WSe_2_ FET (Device E). From the backward *I*_D_–*V*_GS_ sweeps in Figure 4a, we extracted four key metrics: the maximum drain current *I*_max_, extrinsic field-effect mobility *μ*_FE_, subthreshold swing *SS*, and threshold voltage *V*_T_. Below, we highlight how two key metrics, *I*_max_ and *μ*_FE_, evolve with different channel structures. The microribbon FET had an *I*_max_ of 5.9 μA μm^−1^ and a *μ*_FE_ of 12 cm^2^ V^−1^ s^−1^, the passivated-edge nanoribbon FET had an *I*_max_ of 43 μA μm^−1^ and a *μ*_FE_ of 47 cm^2^ V^−1^ s^−1^, whereas the open-edge nanoribbon FET had an *I*_max_ of 1.6 μA μm^−1^ and a *μ*_FE_ of 2 cm^2^ V^−1^ s^−1^.

Consistent with previous reports,^[9,12,13,52,53]^ the open-edge nanoribbon FET exhibited degraded *I*max and *μ*_FE_ compared to the microribbon FET, due to a more prominent role of edge disorder in nanoribbons, and increased densities of defects and charge traps from nanolithography and etching. In contrast, the passivated-edge nanoribbon FET outperformed the microribbon FET with improved *I*_max_ and *μ*_FE_. Compared to the open-edge nanoribbon FET, the passivated-edge nanoribbon FET exhibited significant enhancements: a 30-fold increase in *I*_max_, and a 23-fold increase in *μ*_FE_.

As control experiments, Figure S12, Supporting Information plots the FET transport in monolayer WO_x_Se_y_ and shows that the amorphous WO_x_Se_y_ is insulating. Additionally, Figure S13, Supporting Information plots the transport in a WSe_2_ microribbon FET before and after a KOH bath, showing only a slight change in performance (21% decrease in *I*_max_) compared with the enhancements from edge passivation. Thus, the KOH etch process does not induce significant disorder or doping in WSe_2_ or affect the Pd/Au contacts. Moreover, the probable impact of KOH on the nanoribbon surface should be minimal since the surface of the WSe_2_ nanoribbons was protected by PMMA nanoribbons on top.

Above all, we ascribe the overall improvement in *I*_max_ of the passivated-edge nanoribbon FET over the open-edge nanoribbon FET to two factors: increased p-doping, and reduced edge scattering. Both charge transfer from high-work-function WO_x_Se_y_ at the edges to the nanoribbon channel^[54]^ and the filling in chalcogen vacancies with oxygen atoms^[31,45]^ enhance p-doping. Additionally, passivating the dangling bonds and Se vacancies at the edges reduces carrier scattering.^[31,32]^ We accounted for the change in doping by extracting *I*_D_ under strong inversion at a constant over-drive voltage *V*_GS_ – *V*_T_ = −50 V (corresponding to a constant hole concentration of 3.8 × 10^12^ cm^−2^) as shown in Figure S14, Supporting Information, yielding values of 32 and 1.6 μA μm^−1^ for passivated-edge and open-edge nanoribbons, respectively. Consequently, reduced edge scattering accounted for 74% of the increase in *I*_max_, while edge doping contributed the remaining 26%. The notable enhancement in the electrical performance of the passivated-edge nanoribbon FET primarily arises from reduced edge disorder, aligning with the conclusions drawn from the PL study.

Figure 4b,c exhibits a linear and symmetric dependence of *I*_D_ on *V*_DS_ for the palladium-contacted WSe_2_ microribbon FET and the passivated-edge nanoribbon FET, respectively. Figure 4d shows a nonlinear and asymmetric dependence of *I*_D_ on *V*_DS_ for the open-edge nanoribbon FET. The linear output characteristics in Figure 4b,c suggest ohmic contacts at room temperature for both microribbon and passivated-edge nanoribbon FETs. The nonlinear output characteristics in Figure 4d suggest a large Schottky barrier height for the open-edge nanoribbon FET. Since the contacts on the original FET did not change after KOH etch, we hypothesize that a Schottky junction was created between each pair of the microribbon under the source/drain contact and the nanoribbon channel. Our interpretation for this behavior is that the Fermi level of open-edge WSe_2_ nanoribbons is higher than that of microribbons. This is likely because etching leads to the formation of Se vacancies at the open edges, as shown in Figure S8, Supporting Information, thereby reducing the level of p-doping in these edges compared to the bulk channel.^[45,55]^ In addition, variations in the edge structure and width of the nanoribbon near the source and drain might give rise to distinct local doping concentrations, leading to distinct Schottky junction widths and hence asymmetric output curves in open-edge nanoribbon FETs. Conversely, passivated-edge nanoribbon FETs exhibit improved p-doping, which reduces Schottky junction widths on both sides. Narrow Schottky junction width facilitates hole injection into the contacts on both sides, potentially resulting in more symmetric output curves in passivated-edge nanoribbon FETs.

Figure S15, Supporting Information shows the transfer curves of additional FETs (Devices A–D and F–G) at each stage of fabrication. Tables S1,S2, and S3, Supporting Information summarize the dimensions and metrics of Devices A–G with three different channel structures: microribbon, passivated-edge nanoribbon, and open-edge nanoribbon, respectively. Figure S16, Supporting Information demonstrates good cycle-to-cycle stability of passivated-edge nanoribbon FETs.

To further exclude non-ideal effects from contact resistance, we performed four-probe measurements on a microribbon FET (*W*= 5 μm) and a passivated-edge nanoribbon FET (*W* = 55 nm), as shown in Figure S17, Supporting Information. The passivated-edge nanoribbon exhibited a conductivity mobility *μ*_con_ of ≈40 cm^2^ V^−1^ s^−1^, significantly higher than that of the microribbon of ≈15 cm^2^ V^−1^ s^−1^. This improvement in *μ*_con_ confirms that oxidation-based edge passivation enhances the hole transport of WSe_2_ in the nanoribbon limit.

**Figure 5.**
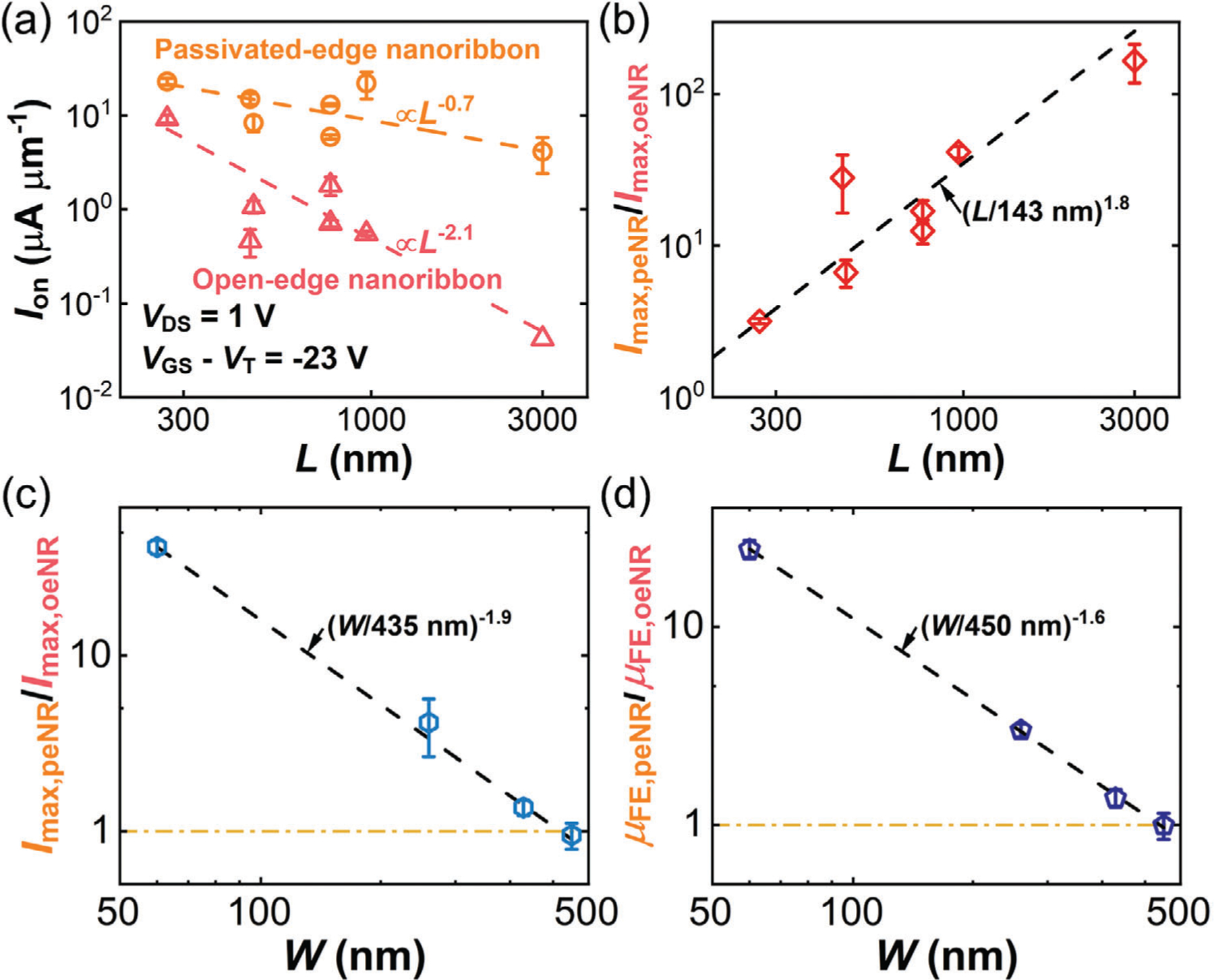
Channel length and width scaling. a) *I*_on_ versus *L* for both passivated-edge and open-edge nanoribbon FETs (Devices A–G) at *V*_DS_ = 1 V and *V*_GS –_ *V*T = −23 V. The dashed lines are power-law fits to the data, showing *I*_on_ scales with *L* as *L*^−0.7^ for passivated-edge nanoribbon FETs (Pearson’s *r*^2^ = 0.27), and *L*^−2.1^ for open-edge nanoribbon FETs (*r*^2^ = 0.90). b) The ratio of *I*_max_ for passivated-edge nanoribbon FETs to open-edge counterparts (*I*_max,peNR_/*I*_max,oeNR_) versus *L*. The dashed line is a power-law fit to the data, showing *I*_max,peNR_/*I*_max,oeNR_ = (*L*/143 nm)^1.8^ with *r*^2^ = 0.97. c) *I*_max,peNR_/*I*_max,oeNR_ versus *W* at *V*_DS_ = 1 V. The dashed line is a power-law fit to the data, showing *I*_max,peNR_/*I*_max,oeNR_ = (*W*/435 nm)^−1.9^ with *r*^2^ = 0.9994. d) The ratio of *μ*_FE_ for passivated-edge nanoribbon FETs to open-edge counterparts (*μ*_FE,peNR_/*μ*_FE,oeNR_) versus *W*. The dashed line is a power-law fit to the data, showing *μ*_FE,peNR_/*μ*_FE,oeNR_ = (*W*/450 nm)^−1.6^ with *r*^2^ = 0.9996. The error bars include cycle-to-cycle variability.

Since WO_x_Se_y_ edge passivation improves the hole conduction near the channel edges, its effect is expected to be more prominent in both longer and narrower channels. To validate this hypothesis, we analyzed the electrical performance of two sets of FETs in Figure 5 as a function of *L* and *W*, respectively: One set (Devices A–G) has similar *W* of 40–70 nm but varying *L* from 270 to 3000 nm (Figure 5a,b). The other set (Devices H–J in Figure S18, Supporting Information and Device E) has similar *L* of 930– 970 nm but varying *W* from 60 to 460 nm (Figure 5c,d).

Figure 5a shows the dependence of on-current *I*_on_ on *L*, extracted at *V*_DS_ = 1V and *V*_GS –_ *V*T = −23 V. Figure S19, Supporting Information shows *I*_on_ versus *W*. *I*_on_ scales as *L*^−0.7^ and *W*^−1.4^ for passivated-edge nanoribbon FETs, and *L*^−2.1^ and *W*^0.5^ for open-edge nanoribbon FETs. In comparison, the Drude model predicts the current density scales as *L*^−1^ and *W*^0^ for constant carrier density and mobility. The near inverse length scaling of current density indicates that hole transport in passivated-edge nanoribbons is channel-dominated in the regime of *L* > 270 nm and *W* = 40–70 nm, whereas the higher length scaling indicates hole transport in open-edge nanoribbons is limited by edge disorder in the same regime. We hypothesize the deviation from *L*^−1^ scaling for the passivated-edge nanoribbon FETs is due to the convolution of contact resistance. The decreasing current density with increasing width in passivated-edge nanoribbon FETs suggests that heavily p-doped edges dominate hole transport over the bulk, whereas the increasing current density with increasing width in open-edge nanoribbon FETs suggests degraded hole conductivity at the open edges compared to the bulk.

To analyze the overall improvement in FET transport from edge passivation, Figure 5b plots the ratio of *I*_max_ for passivated-edge nanoribbon (peNR) FETs to their open-edge nanoribbon (oeNR) counterparts (*I*_max,peNR_/*I*_max,oeNR_) versus *L*. Figure 5c,d shows *I*_max,peNR_/*I*_max,oeNR_ and *μ*_FE,peNR_/*μ*_FE,oeNR_ versus *W*, respectively. The differences in the electrical performance of passivated-edge and open-edge nanoribbon FETs become more pronounced for longer and narrower channels. For 40–70 nm wide nanoribbons, the electrical performance of the passivated-edge and open-edge nanoribbons converges at *L* ≲143 nm, and similarly for ≈1 μm long nanoribbons, the performance of passivated-edge and open-edge nanoribbons converges at *W* ≳ 450 nm. These scaling values suggest that the performance of 40–70 nm wide nanoribbon FETs is contact-limited and unaffected by edge passivation at *L* ≲143 nm (or *L/W* < 2–3), and that the edge transport becomes insignificant compared to the bulk transport at *W* ≳ 450 nm. Given that the contact resistance in this work (≈100 kΩ μm shown in Figure S17, Supporting Information) is about two orders of magnitude higher than the state of the art for monolayer WSe_2_,^[5G]^ we expect the edge passivation effect to remain prominent for ≲70 nm wide nanoribbon FETs with improved contacts even in the sub-10 nm channel length regime.

**Figure 6.**
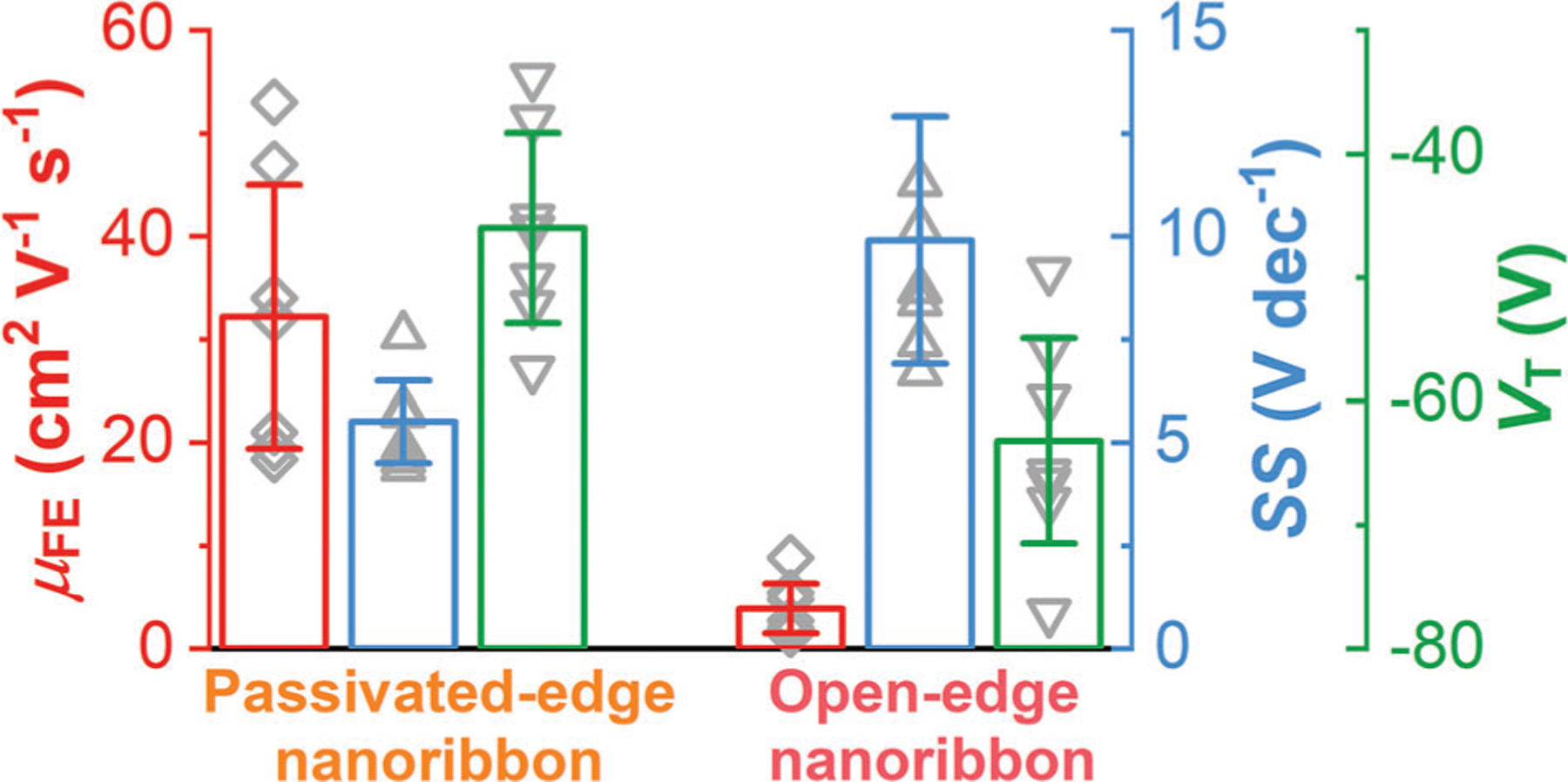
Statistical analysis of key metrics of seven nanoribbon FETs (Devices A–G) with WO_x_Se_y_ passivated edges versus with open edges.

Three key metrics of nanoribbon FETs (Devices A–G), including *μ*_FE_, *SS*, and *V*_T_, exhibit no dependence on channel dimensions within their range. Therefore, we analyze the statistics of these metrics for passivated-edge versus open-edge nanoribbon FETs in Figure 6. On average, passivated-edge nanoribbon FETs exhibited a *μ*_FE_ of 32 ± 13 cm^2^ V^−1^ s^−1^, a *SS* of 5.5 ± 1.0 V dec^−1^, and a *V*_T_ of −46.0 ± 7.7 V. In comparison, open-edge nanoribbon FETs exhibited a *μ*_FE_ of 4 ± 2 cm^2^ V^−1^ s^−1^, a *SS* of 9.9 ± 3.0 V dec^−1^, anda *V*T of −63.2 ± 8.3 V. The application of WO_x_Se_y_ edge passivation to these nanoribbon FETs increased *μ*_FE_ by an average of 10 ± 6 times, decreased *SS* by 40 ± 15%, and increased *V*_T_ by 17.2 ± 6.7 V. From the shift in *V*_T_, the hole doping from edge passivation was (1.3 ± 0.5) × 10^12^ cm^−2^, which is one order of magnitude smaller than that from surface doping with WO_x_Se_y_ on top of WSe_2_.^[36]^

Figure S20, Supporting Information compares the hysteresis in passivated-edge and open-edge nanoribbon FETs (Devices A– G). On average, the passivated-edge nanoribbon FETs exhibited a hysteresis of 27 ± 6 V, while the open-edge nanoribbon FETs showed a hysteresis of 31 ± 9 V. The observed 4 V increase in hysteresis after KOH etch translates to an increased trap density of 3 × 10^11^ cm−2, equating to 0.18 traps per nanometer along a 60 nm wide nanoribbon. The substantial hysteresis in the nanoribbon FETs primarily originates from the hysteresis in their microribbon counterparts, which have an average hysteresis of 23 ± 4 V.

**Figure 7.**
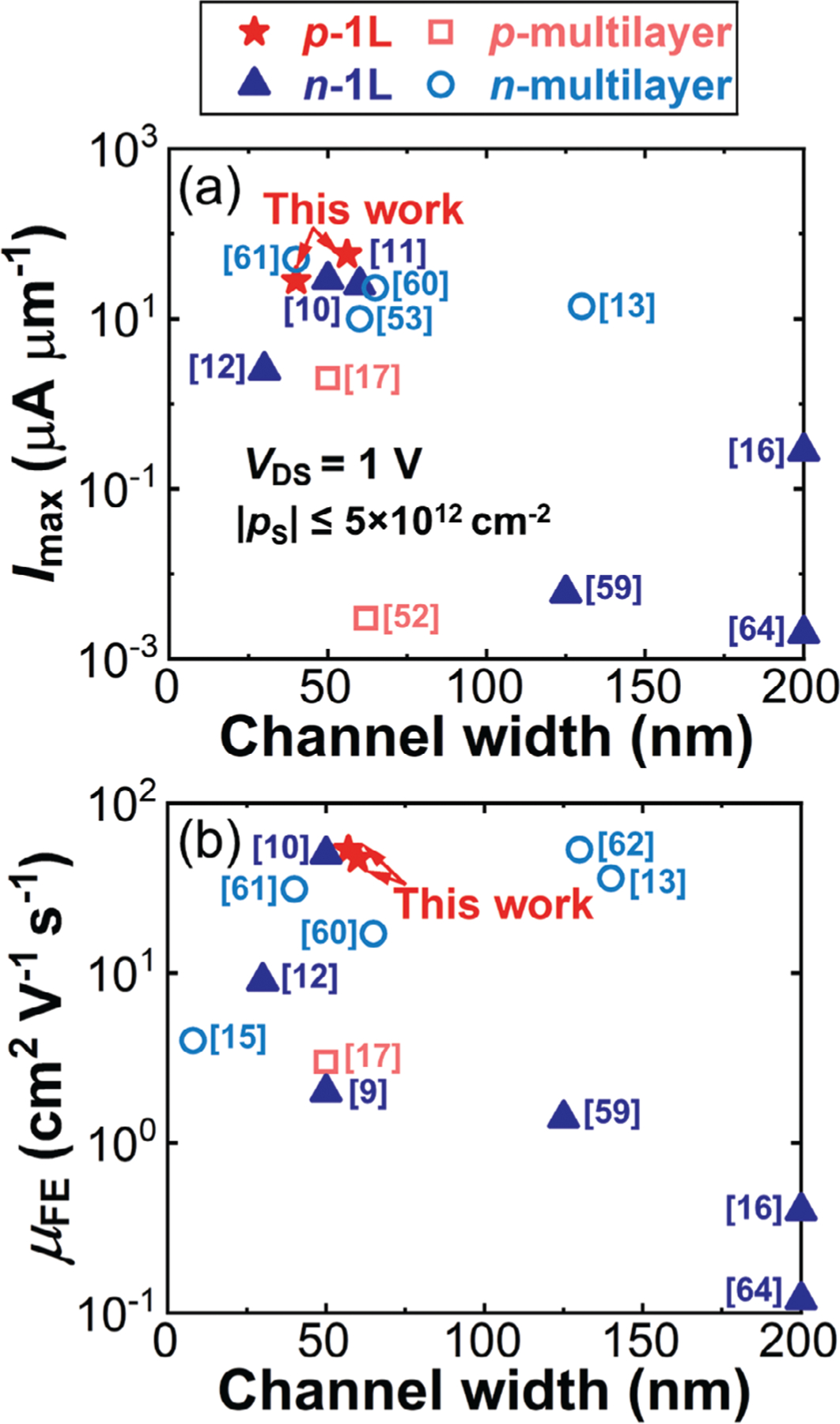
Benchmarking monolayer WSe_2_ nanoribbon FETs with WO_x_Se_y_ passivated edges against other TMD nanoribbon FETs. a) Benchmarking *I*_max_ versus the channel width. *V*_DS_ = 1 V, and |*p*_S_| ≤ 5 × 10^12^ cm^−2^. b) Benchmarking *μ*_FE_ versus the channel width.

Finally, Figure 7 benchmarks monolayer WSe_2_ nanoribbon p-FETs with WO_x_Se_y_ passivated edges against other reported TMD nanoribbon FETs summarized in Table S4, Supporting Information.^[9–17,52,53,57–64]^ Figure 7a shows the benchmarking of *I*_max_ at *V*_DS_ = 1 V against the channel width. To ensure a fair comparison, the upper limit of the carrier density near the source *p*_S_ was set to 5 × 10^12^ cm^−2^. *I*_max_ is a reliable metric of assessing and benchmarking 2D transistors, because it is directly measured with minimum derivations and associated uncertainties.^[65]^ However, some studies had very different channel lengths,^[12,17,59]^ did not reach |*p*S| ≈5 × 10^12^ cm^−2^,^[9,10,15,16,52,58]^ or measured transfer curves at *V*_DS_ < 1 V.^[9,15,61]^ To facilitate comparison with these studies, a second benchmark plot on *μ*_FE_ versus channel width was constructed in Figure 7b.

Among the reported TMD nanoribbon FETs, the best TMD nanoribbon n-FET achieved an *I*_max_ of 50 μA μm^−1^ at *V*_DS_ = 1 V and a *μ*_FE_ of 31 cm^2^ V^−1^ s^−1^, with a channel width of 40 nm and a channel thickness of 6 nm.^[60]^ On the other hand, the leading TMD nanoribbon p-FET had an *I*_max_ of 2 μA μm^−1^ at *V*_DS_ = 1 V and a *μ*_FE_ of 3 cm^2^ V^−1^ s^−1^, with a channel width of 50 nm and a channel thickness of 4.9 nm.^[17]^ It is obvious that the performance of TMD nanoribbon p-FETs significantly lags behind that of n-FETs, presumably because chalcogen vacancies at the edges n-dope the nanoribbon channels and degrade hole mobility.^[45,66]^ In comparison, Device D in this work, a monolayer p-FET with a channel width of 57 nm, achieved an *I*_max_ of 49 μA μm^−1^ at *V*_DS_ = 1 V with a *μ*_FE_ of 53 cm^2^ V^−1^ s^−1^. Therefore, monolayer WSe_2_ nanoribbon p-FETs with WO_x_Se_y_ passivated edges demonstrated comparable electrical performance with the state-of-the-art TMD nanoribbon n-FETs, effectively bridging the performance gap between p-type and n-type TMD nanoribbons.

## 3. Conclusion

We demonstrated a facile edge passivation method that significantly enhances the electrical performance of WSe_2_ nanoribbon p-FETs. We achieved this by fabricating monolayer WSe_2_ nanoribbon transistors with amorphous WO_x_Se_y_ passivated edges using nanolithography and a controlled remote O_2_ plasma process. WO_x_Se_y_ edge passivation significantly reduces edge disorder and enhances the material quality of WSe_2_ nanoribbons, while lightly p-doping the nanoribbons.

Looking ahead, due to its simplicity and effectiveness, this edge passivation method could potentially be incorporated into existing CMOS fabrication, paving the way for integrating high-performance, ultra-scaled WSe_2_ p-FETs within commercial silicon foundries. While WO_x_Se_y_ is not stable at 200 °C, it could be converted to WO_3_ during atomic layer deposition,^[41]^ which still fulfills the role of dangling-bond and vacancy passivation. Multiple strategies can be implemented in conjunction with edge passivation to enhance the electrical performance of monolayer WSe_2_ nanoribbon p-FETs, such as lowering the contact resistance with p-type van der Waals (vdW) contacts,^[67]^ reducing interface disorder with vdW dielectric-semiconductor interfaces,^[68]^ and improving the material quality of WSe_2_.^[69]^ Furthermore, this oxidation-based edge passivation approach can be extended to other TMD nanoribbon p-FETs, since both WO_x_ and MoO_x_ are stable solid-state p-dopants.^[70]^

## 4. Experimental Section

### STEM Sample Fabrication:

First, exfoliated monolayer WSe_2_ was transferred onto holey TEM grids. Second, EBL was used to pattern PMMA nanoribbons on the freestanding monolayer WSe_2_. Third, remote O_2_ plasma (Tergeo plasma cleaner, PIE scientific; 50 W, 20 s, 0.5 sccm O_2_; the same below) was used to oxidize exposed monolayer WSe_2_. Fourth, the PMMA mask was removed using chloroform. Finally, the samples were annealed at 200 °C (unless otherwise noted) in vacuum overnight before loading into the STEM column, to minimize carbon deposition during atomic resolution imaging.

### Nanoribbon Fabrication for Raman and PL:

First, a WSe_2_ flake was transferred onto a SiO_2_/Si substrate. Then atomic force microscopy (AFM) tip-based cleaning was used to remove polymer residues on monolayer WSe_2_.^[71]^ Next, tDPN was used to deposit an array of PMMA nanoribbons of varying widths onto monolayer WSe_2_.^[12,37]^ In tDPN, a heated AFM tip writes a molten ink onto a substrate.^[72]^ The temperature gradient between the tip and the substrate controls the ink flow.^[73]^ The deposited PMMA served as an etch mask in subsequent steps. Afterward, remote O_2_ plasma was used to convert the un-masked WSe_2_ into WO_x_Se_y_, forming passivated-edge monolayer WSe_2_ nanoribbons. Finally, the sample was immersed in a KOH bath (1 M) for 10 s to remove WO_x_Se_y_,^[34]^ followed by de-ionized (DI) water rinse, forming open-edge monolayer WSe_2_ nanoribbons.

### Transistor Fabrication:

First, a WSe_2_ flake obtained by gold-assisted large-area exfoliation^[74]^ was transferred onto a 285 nm SiO_2_/Si substrate. Subsequently, the monolayer region of the WSe_2_ flake was patterned into a 5-μm-wide ribbon using e-beam lithography and XeF_2_ etch.^[75]^ The contact electrodes consisting of 5 nm Pd/50 nm Au were then deposited onto the monolayer WSe_2_ using e-beam lithography, e-beam evaporation, and liftoff, forming a microribbon FET. Pd was chosen to contact monolayer WSe_2_ because Pd is a high-work-function metal that results in a low hole Schottky barrier to facilitate hole transport.^[33,67,76]^ Afterward, AFM tip-based cleaning was used to remove polymer residues on the microribbon,^[71]^ which improves the homogeneity of subsequent nanoribbon FETs. Next, tDPN was used to deposit a PMMA nanoribbon onto the microribbon FET. Then remote O_2_ plasma was used to convert the exposed WSe_2_ into WO_x_Se_y_, forming a passivated-edge monolayer WSe_2_ nanoribbon FET. Finally, the sample was immersed in a KOH bath (1 M) for 10 s to remove WO_x_Se_y_,^[34]^ followed by DI water rinse, forming an open-edge monolayer WSe_2_ nanoribbon FET.

### STEM Measurements:

The samples were imaged in an aberration corrected STEM (Themis Z, Thermo Fisher Scientific). The STEM was operated at 300 kV at a semi-convergence angle of 18 mrad with a beam current of 50 pA. Elemental maps were collected with a Super-X EDS detection system with a beam current of 200 pA.

### Raman and PL Measurements:

Raman and PL measurements were performed on a confocal Raman microscope (Nanophoton Raman 11) using a 532 nm laser with a 100× objective. Raman spectra were obtained using a grating of 2400 l mm^−1^. PL spectra were obtained using a grating of 600 l mm^−1^. Both passivated-edge and open-edge WSe_2_ nanoribbons were measured with PMMA nanoribbons on top, to ensure consistent dielectric environment of the nanoribbon FETs.

### Electrical Measurements:

All the electrical measurements were performed at room temperature in air using a semiconductor parameter analyzer (Agilent, 4155C). For the nanoribbon measurements, the PMMA mask was left on top, as PMMA provides a well-defined and consistent dielectric environment with a low density of charge traps and mild (below 10^12^ cm^−2^) p-doping.^[77,78]^ Microribbon transistors were measured without passivation.

## Supplementary Material

Supinfo

## Data Availability

The data that support the findings of this study are available from the corresponding author upon reasonable request.
