# Supplementary material for: Edge-Passivated Monolayer WSe2 Nanoribbon Transistors
Source: Adv Mater. Author manuscript; Available in PMC 2025 Sep 1. (PMC11436303; doi:10.1002/adma.202313694)
Supplement: Supinfo [file NIHMS2009834-supplement-Supinfo.pdf]

Supporting Information

Edge-Passivated Monolayer WSe_2 Nanoribbon Transistors

Sihan Chen,[†,*] *Yue Zhang,*[‡] *William P. King,*[†,‡,#] *Rashid Bashir,*[†,§,*] *Arend M. van der Zande*[†,‡,#,*]

[†]Holonyak Micro and Nanotechnology Laboratory, The Grainger College of Engineering, University of Illinois Urbana-Champaign, Urbana, Illinois 61801, United States

[‡]Department of Mechanical Science and Engineering, The Grainger College of Engineering, University of Illinois Urbana-Champaign, Urbana, Illinois 61801, United States

[#]Materials Research Laboratory, The Grainger College of Engineering, University of Illinois Urbana-Champaign, Urbana, Illinois 61801, United States

[§]Department of Bioengineering, The Grainger College of Engineering, University of Illinois Urbana-Champaign, Urbana, Illinois 61801, United States

*Emails: schen141@illinois.edu; rbashir@illinois.edu; arendv@illinois.edu

1. Raman and Photoluminescence of monolayer WSe_2 before and after oxidation

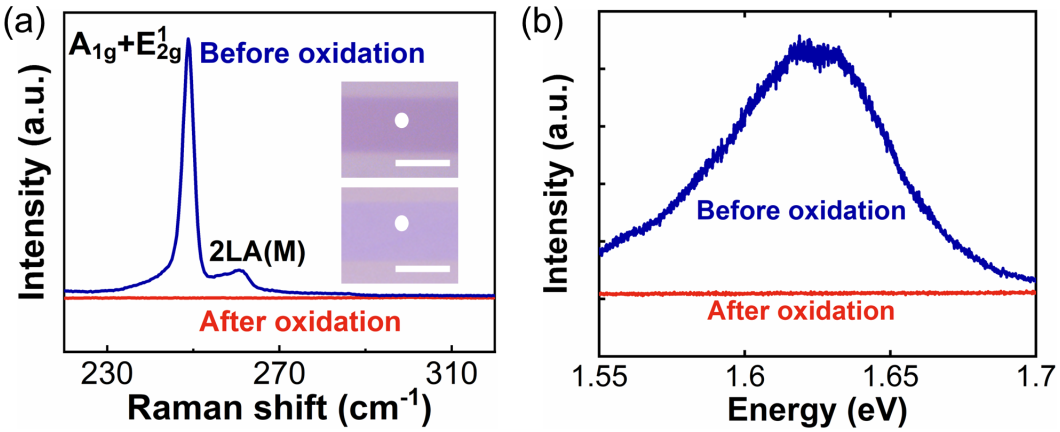

Figure S1. (a) Typical Raman spectra of monolayer WSe_2 before and after oxidation. Insets: Optical images of the monolayer WSe_2 microribbon used for Raman and photoluminescence (PL) characterization before (top) and after (bottom) oxidation, where the white dots indicate the location of the measurements. Scale bar, 5 μm. (b) Typical PL spectra of monolayer WSe_2 before and after oxidation.

2. Transistor fabrication

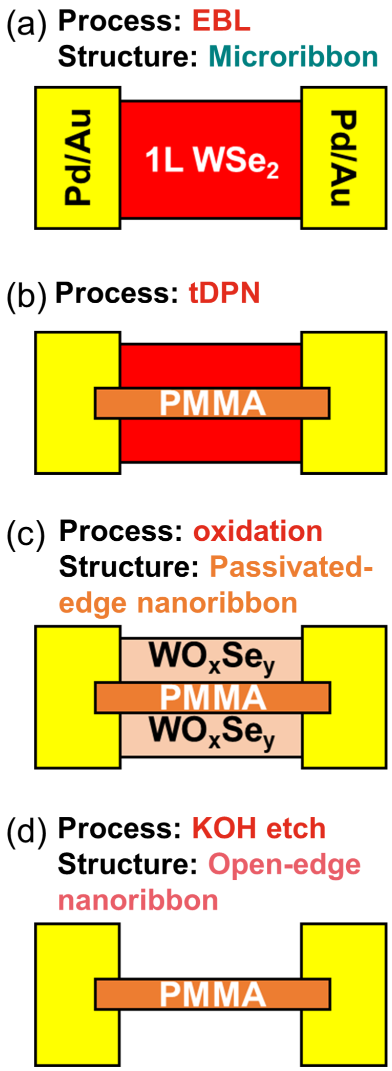

Figure S2. (a) Fabrication of a monolayer WSe_2 microribbon field-effect transistor (FET) using electron beam lithography (EBL). (b) Deposition of a PMMA nanoribbon mask onto a microribbon FET using thermal dip-pen nanolithography (tDPN).[1,2] (c) Oxidation of un-masked monolayer WSe_2 using remote O_2 plasma. (d) Removal of WO_xSe_y using KOH etch.[3]

3. STEM sample fabrication

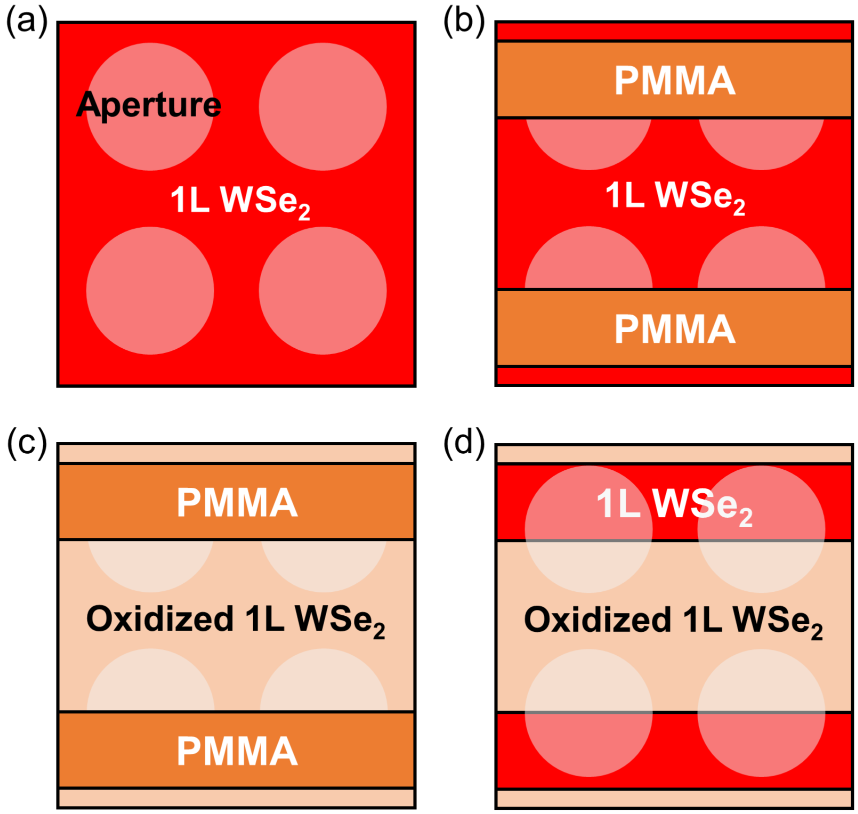

Figure S3. Schematic illustration of (a) transfer of exfoliated monolayer WSe_2 onto holey TEM grids, (b) fabrication of PMMA nanoribbons on the freestanding monolayer WSe_2 using EBL, (c) oxidation of un-masked monolayer WSe_2 using remote O_2 plasma, and (d) removal of the PMMA mask using solvents, followed by overnight vacuum annealing.

4. Nanoribbon fabrication for Raman and PL

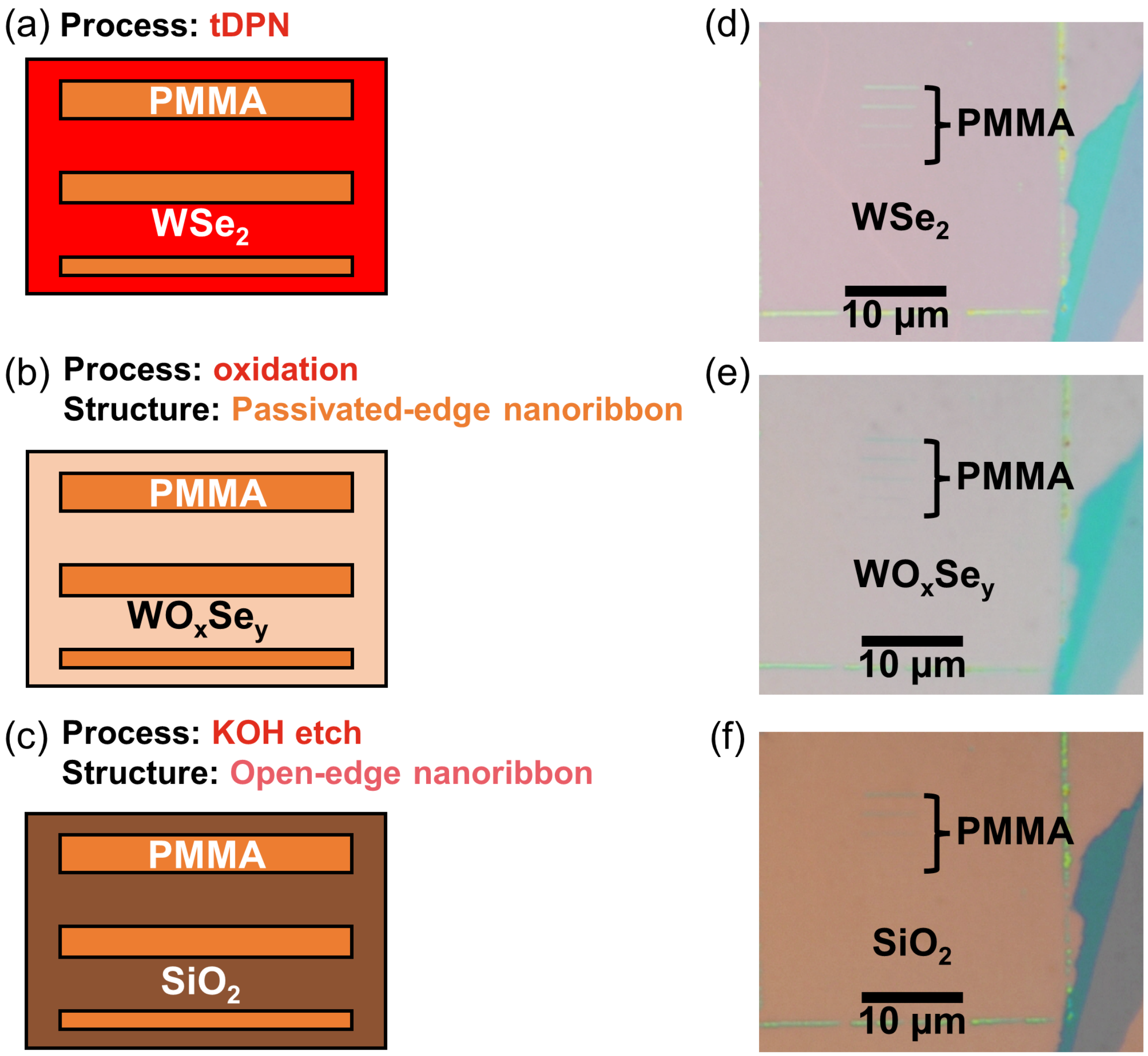

Figure S4. Schematic illustration of (a) deposition of an array of PMMA nanoribbons onto monolayer WSe_2 using thermal dip-pen nanolithography (tDPN),[1,2] (b) oxidation of un-masked monolayer WSe_2 using remote O_2 plasma, and (c) removal of WO_xSe_y using KOH etch.[3] Optical images of (d) an array of PMMA nanoribbons on monolayer WSe_2, (e) passivated-edge WSe_2 nanoribbons with PMMA nanoribbons on top, and (f) open-edge WSe_2 nanoribbons with PMMA nanoribbons on top.

5. EDS maps

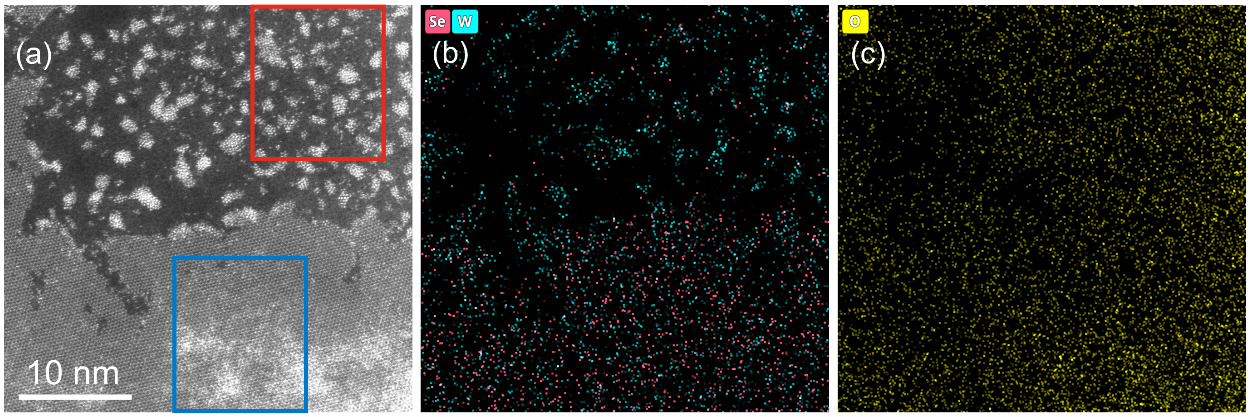

Figure S5. (a) HAADF-STEM image of monolayer WSe_2 with monolayer WO_xSe_y passivated edge. Blue and red rectangles delineate the region used to construct the EDS spectra of WSe_2 and WO_xSe_y in Figure 2b, respectively. EDS maps of (b) W and Se atoms and (c) O atoms that correspond to (a). Annealing does not effectively remove PMMA residues on WSe_2 from nanolithography, leading to a substantial presence of O atoms in the WSe_2 region of the EDS map.

6. Effect of annealing temperature on amorphous monolayer WO_xSe_y at the edge of crystalline monolayer WSe_2

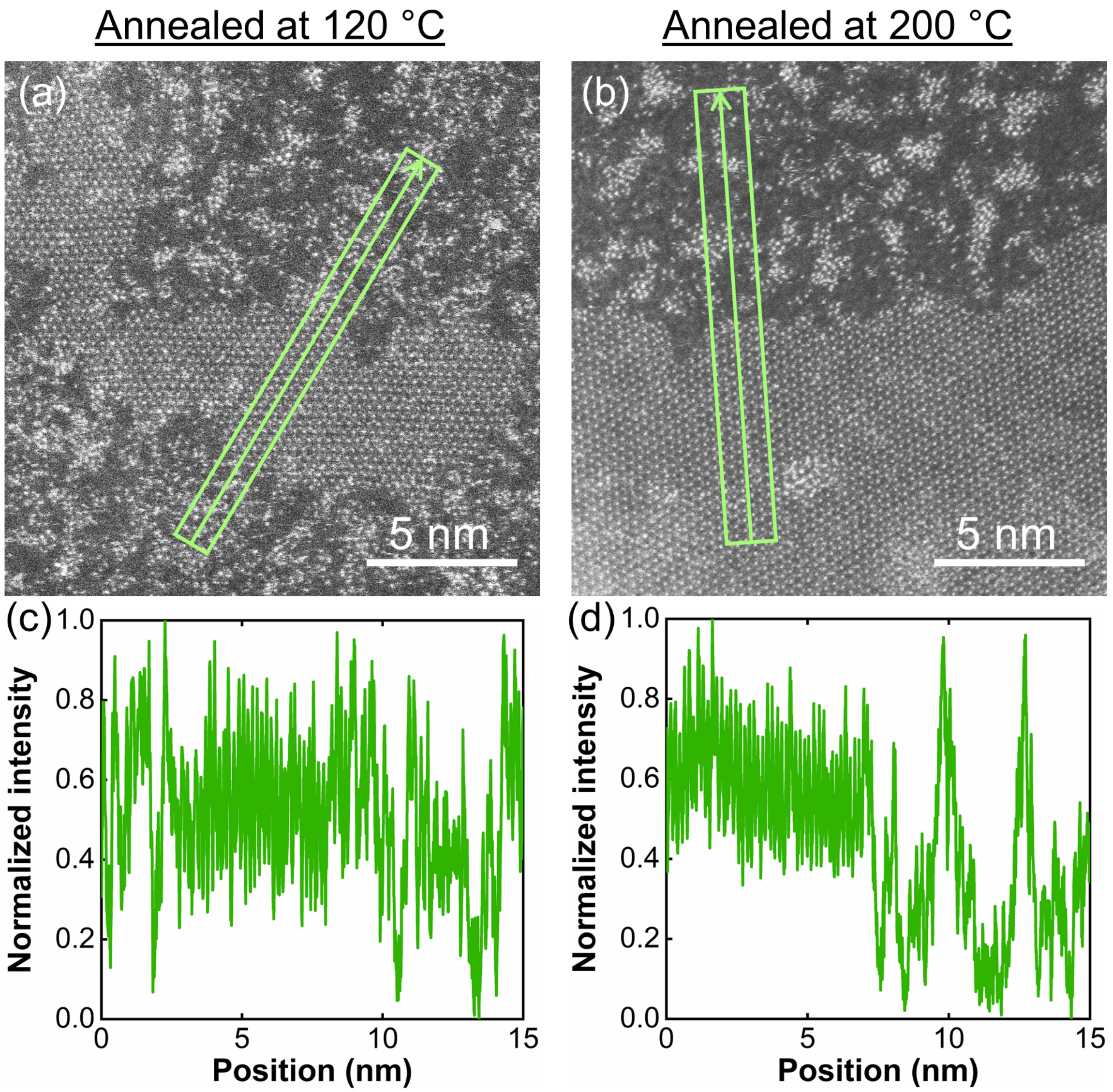

Figure S6. HAADF-STEM images of (a) monolayer WO_xSe_y at the edges of monolayer WSe_2 after annealing at 120 °C in vacuum (10^{-5} Torr), and (b) monolayer WO_xSe_y at the edge of monolayer WSe_2 after annealing at 200 °C in vacuum (10^{-5} Torr). (c) Normalized HAADF-STEM intensity profile along the line in (a). (d) Normalized HAADF-STEM intensity profile along the line in (b).

7. Effect of electron beam irradiation dose on monolayer WO_xSe_y

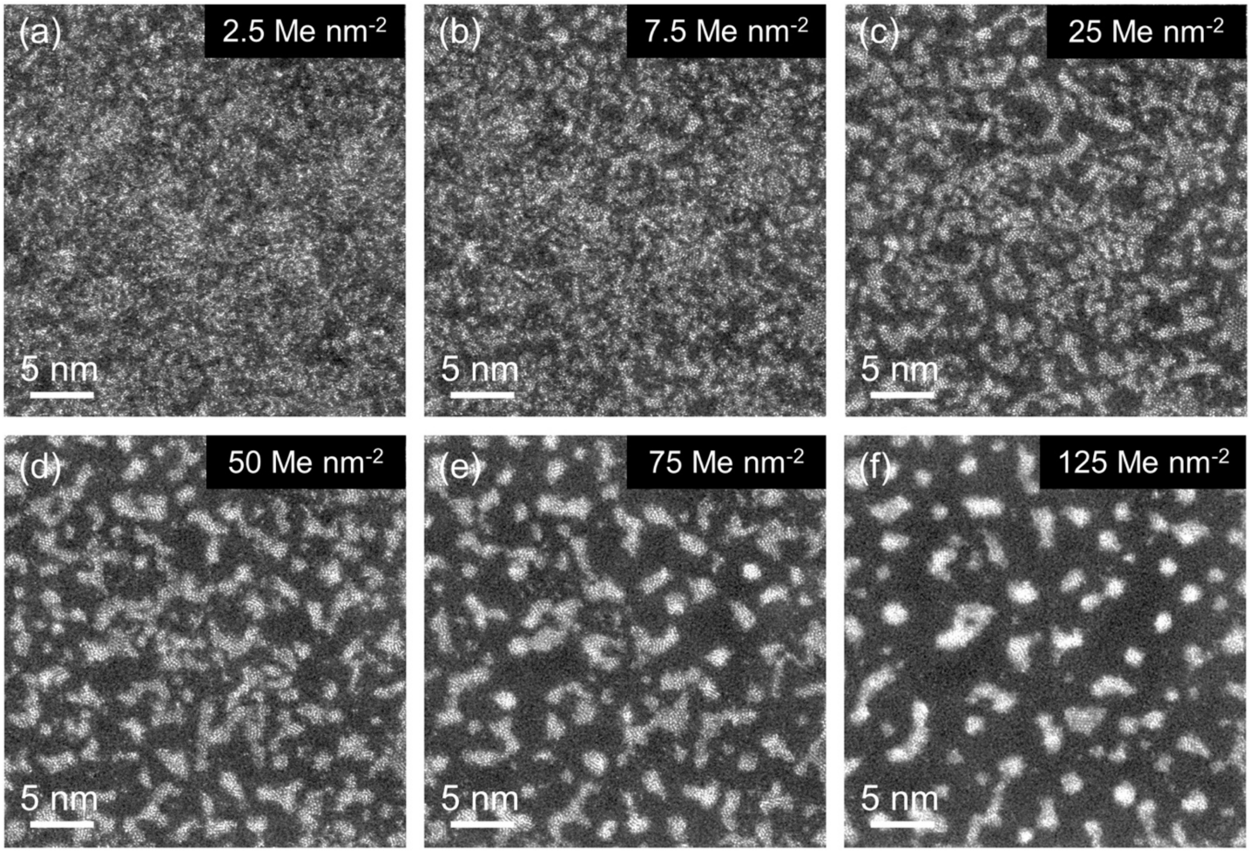

Figure S7. HAADF-STEM images of amorphous monolayer WO_xSe_y with increasing electron beam irradiation doses, from 2.5 Me nm^{-2} up to 125 Me nm^{-2}.

8. HAADF-STEM and EDS images of open-edge monolayer WSe_2 nanoribbons

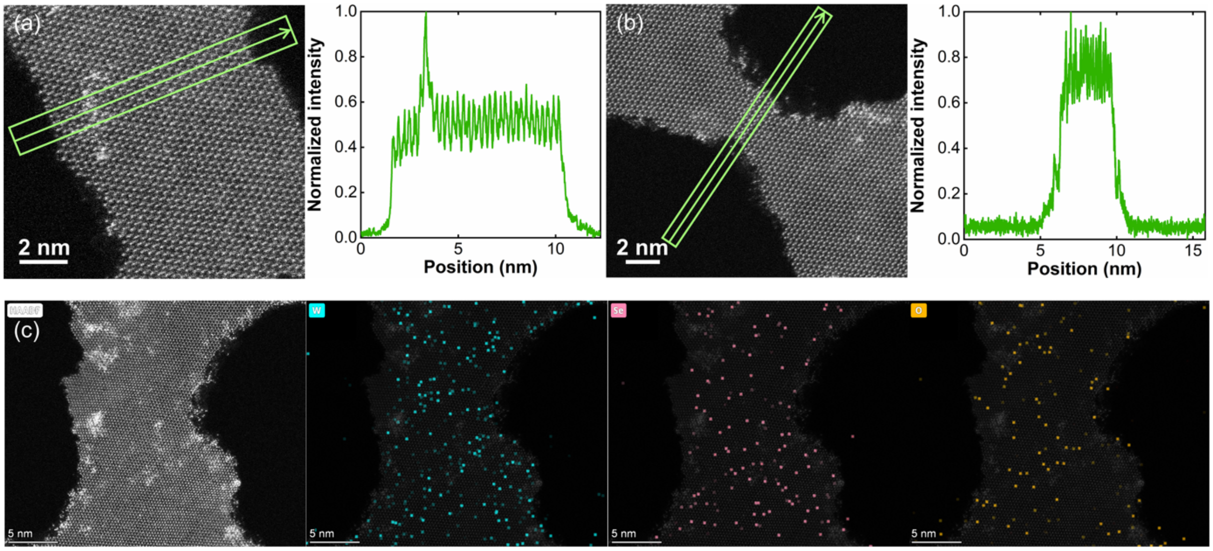

Figure S8. (a) and (b) HAADF-STEM images (left) and corresponding normalized line intensity profiles (right) of two open-edge monolayer WSe_2 nanoribbons following the removal of WO_xSe_y with a KOH bath. HAADF imaging utilizes *Z*-contrast, wherein heavier atoms like tungsten (W: $Z = 74$) appear brighter than lighter atoms like selenium (Se: $Z = 36$). Clearly, the etched edges were predominantly terminated with W atoms. (c) HAADF-STEM image of an open-edge monolayer WSe_2 nanoribbon, and corresponding EDS maps of W, Se, and O atoms overlaid on the HAADF-STEM image. Annealing does not effectively remove PMMA residues on WSe_2 from nanolithography, leading to the presence of O atoms within the WSe_2 region in the EDS map. In contrast, there is no O atom outside the WSe_2 region, confirming that the etched edges are devoid of passivating solid-state materials.

9. Raman microscopy of monolayer WSe_2 nanoribbons

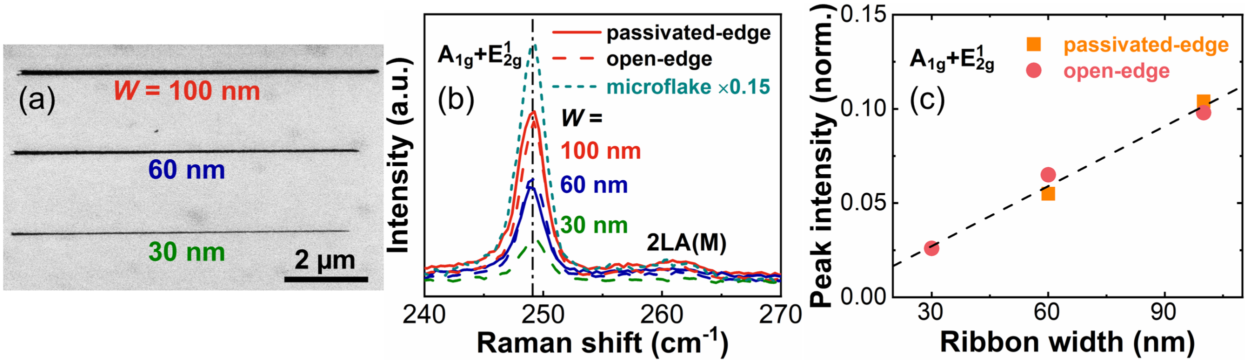

Figure S9. (a) SEM image of the nanoribbons used in the Raman measurements. (b) Raman spectra of three nanoribbons with a width of 100 nm, 60 nm, and 30 nm respectively, comparing the characteristics of both passivated-edge and open-edge nanoribbons. Raman spectrum of the microflake before nanoribbon formation was also plotted for comparison. All passivated-edge and open-edge nanoribbons exhibited identical Raman peak positions (A_{1g}+$E^1{}_{2g}$), confirming the preservation of the crystal structure of monolayer WSe_2 nanoribbons after KOH etching. There was no discernible dependence on ribbon width regarding the Raman peak position, down to a nanoribbon width of 30 nm. For context, it is worth noting that the Raman peaks of monolayer MoS_2 nanoribbons remain consistent with those of microribbons until the ribbon width decreases below 5 nm.[4] (c) Intensities of the A_{1g}+$E^1{}_{2g}$ Raman peak versus ribbon width for both passivated-edge and open-edge nanoribbons. The Raman intensities of nanoribbons are normalized to that of the microflake. The dashed line is a linear fit to data, from which the laser spot size is estimated to be ~ 1.0 μm.

10. PL peak area mapping of open-edge nanoribbons

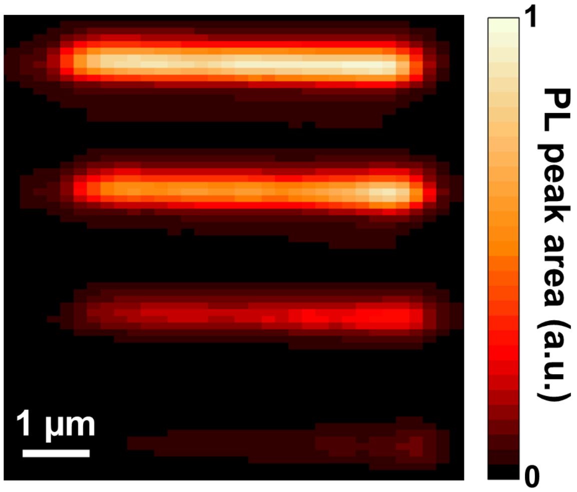

Figure S10. PL peak area map of open-edge nanoribbons, corresponding to the nanoribbons in Figure 3a.

11. Additional data on PL spectra of nanoribbons and width dependence study

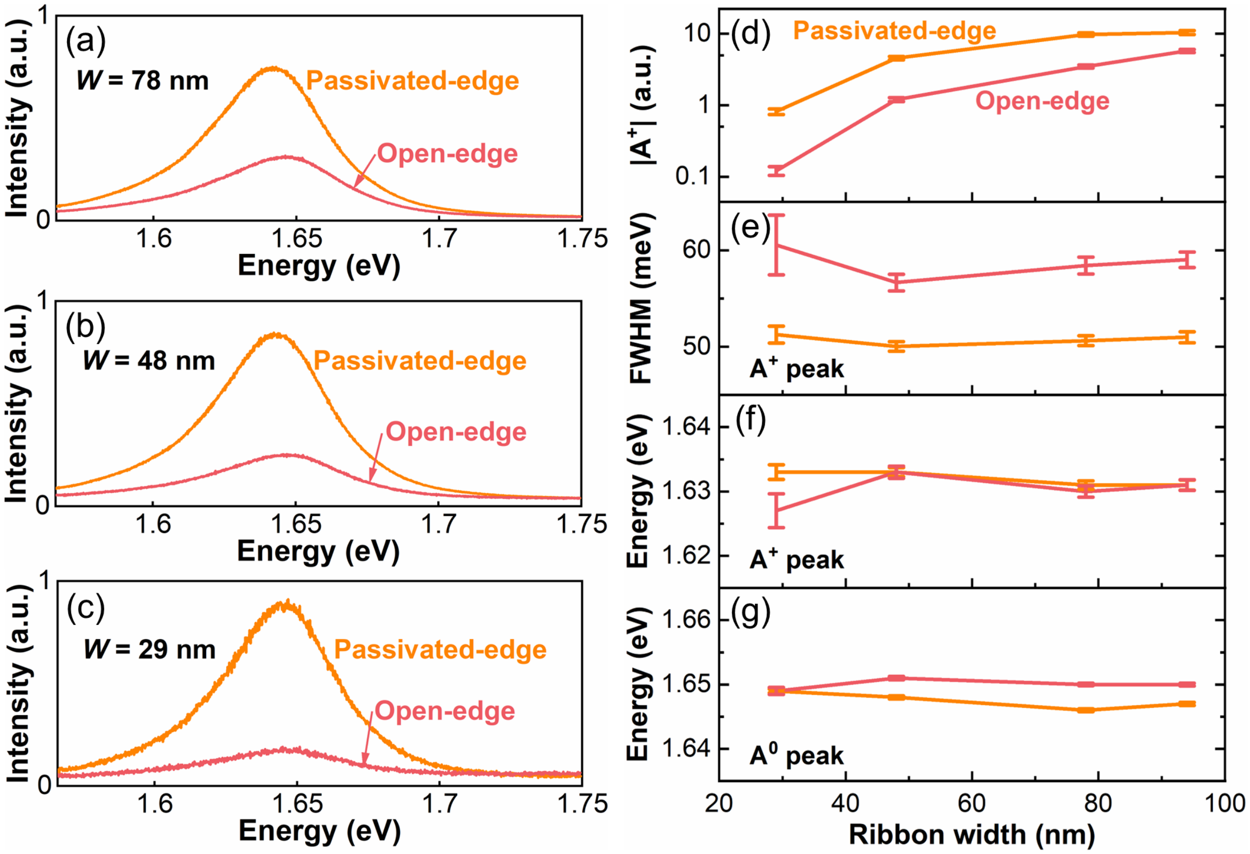

Figure S11. PL spectra of the (a) 78-nm-wide, (b) 48-nm-wide, and (c) 29-nm-wide nanoribbon, comparing the characteristics of passivated-edge and open-edge nanoribbons. Nanoribbon width dependence of (d) the trion peak intensity $|A^+|$, (e) the A^+ peak linewidth (FWHM), the photon energy of (f) the A^+ peak and (g) the A^0 peak, for both edge structures. The intensity of the A^+ peak decreased with ribbon width for both edge structures. The passivated-edge nanoribbons exhibited 81–574% larger trion peak intensities compared to the open-edge nanoribbons, with narrower nanoribbons showing greater difference in the peak intensities. The passivated-edge and open-edge nanoribbons respectively had an average A^+ peak linewidth of 51±1 meV and 59±3 meV. Nanoribbons with different edge structures had similar exciton and trion peak positions, with a maximum difference of 6 meV.

12. Electrical measurements of a monolayer WSe_2 microribbon FET before and after oxidation

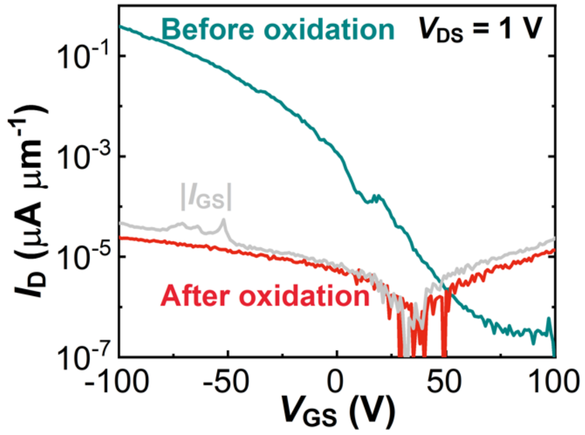

Figure S12. Transfer curves of a monolayer WSe_2 microribbon FET before and after oxidation. The Pd/Au contacted monolayer WSe_2 FET exhibits unipolar hole transport behavior. As a result, we limited all future measurements to a gate bias from +20 V to -100 V, to prevent accidental gate leakage. The WO_xSe_y is not conducting with Pd/Au contacts.

13. Effect of KOH on a monolayer WSe_2 microribbon FET

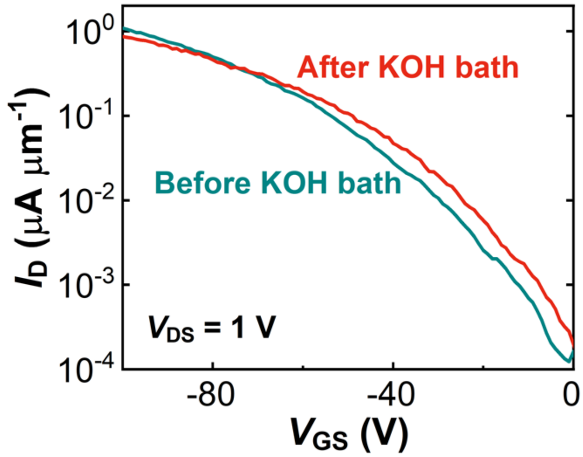

Figure S13. Transfer curves of a monolayer WSe_2 microribbon FET before and after 10 s KOH (1 M) bath. We used de-ionized water rinse and tip-based cleaning to remove KOH traces from the channel surface. The small change observed in the transfer curves after the KOH bath confirms that KOH does not etch WSe_2 or the Pd/Au contacts.

14. I_D versus V_{GS} - V_T for Device E

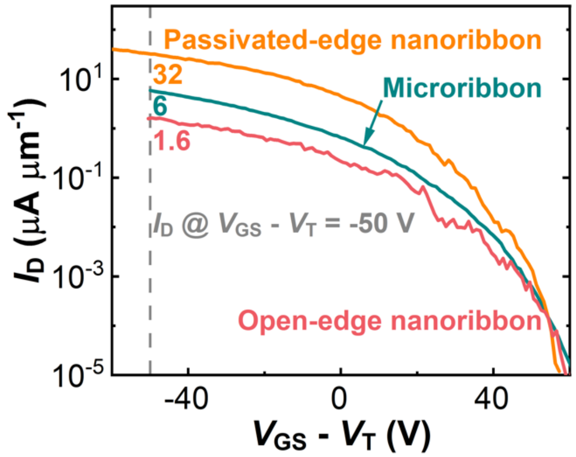

Figure S14. I_D versus V_{GS} - V_T sweeps of a monolayer WSe_2 FET (Device E) as a microribbon, passivated-edge nanoribbon, and open-edge nanoribbon.

15. Transfer curves of other nanoribbon FETs reported in this article

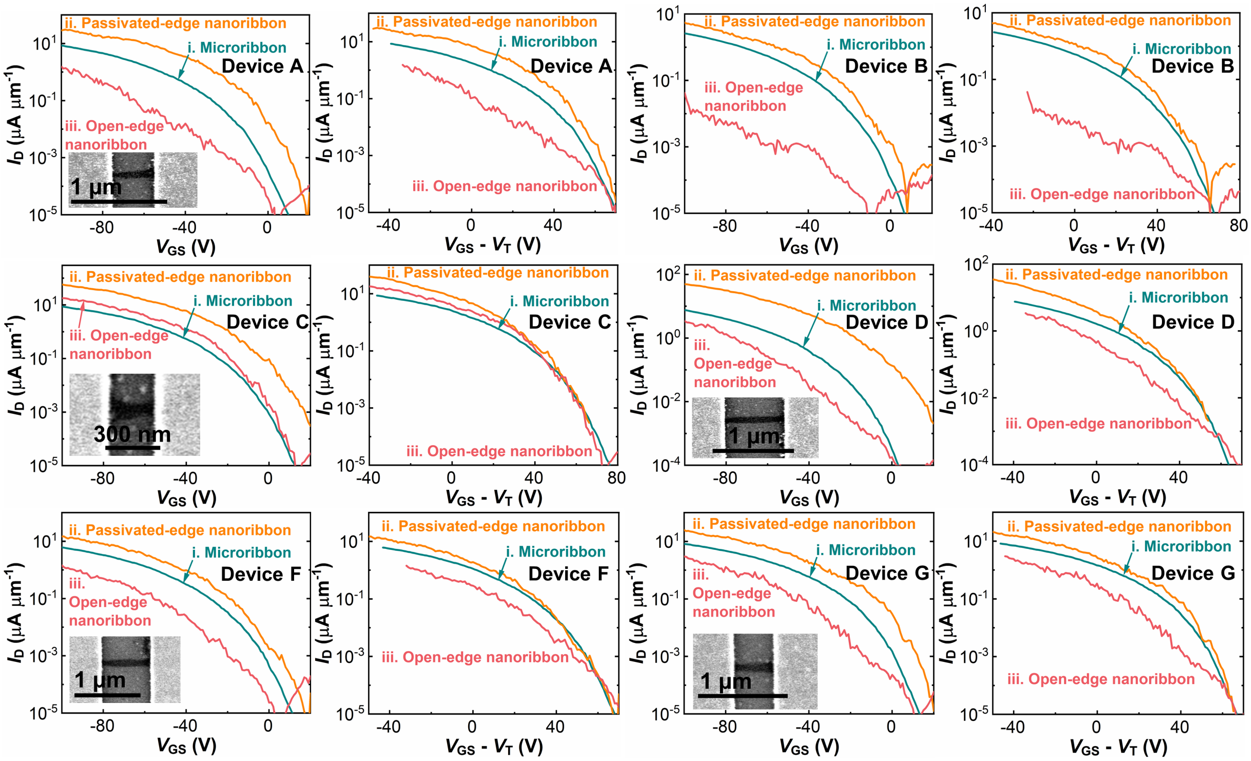

Figure S15. Backward I_D–V_{GS} sweeps of monolayer WSe_2 FETs (Devices A–D and F–G) as microribbons, passivated-edge nanoribbons, and open-edge nanoribbons. V_{DS} = 1 V. Insets: SEM images of the open-edge nanoribbon FETs.

16. Extraction of mobilities and threshold voltage

The extrinsic field-effect mobility μ_{FE} of a field-effect transistor was extracted from a backward I_D–V_{GS} sweep at V_{DS} = 1 V. The mobility was calculated as $\mu_{\mathrm{FE}} = L\mathrm{d}I_{\mathrm{D}}/(W_{\mathrm{eff}}V_{\mathrm{DS}}C_{\mathrm{G}}\mathrm{d}V_{\mathrm{GS}})$, where C_G is 12.1 nF cm^{-2} for 285-nm-thick SiO_2, assuming a parallel-plate capacitor model. Two cancelling factors need to be considered for a more accurate C_G model: (i) fringing fields increase C_G since the nanoribbons are narrow compared to the oxide thickness,[5,6] and (ii) edge states reduce the local density of states and hence the quantum capacitance, limiting fringe effects and lowering C_G.[7–9] The actual C_G will be higher or lower than the parallel-plate model prediction depending on which factor is dominant. Ideally, nanoribbon FETs should be characterized with a sub-5 nm thick gate dielectric to avoid complications from fringe effects. To accommodate the non-uniformity of channel width along the channel length L, an effective channel width W_{eff} was calculated as $W_{\mathrm{eff}} = L/\sum_i(L_i/W_i)$, where $\sum_i(L_i/W_i)$ is the sum of L_i/W_i over all the uniform channel segments i in series. Since hysteresis is present, the mobility determined from the backward I_D–V_{GS} sweep represents a lower limit, whereas the mobility derived from the forward sweep sets an upper limit. For instance, μ_{FE} of a nanoribbon FET with passivated edges (Device D in Table S2) was 53 cm^2 V^{-1} s^{-1} and 164 cm^2 V^{-1} s^{-1}, as extracted from the backward and forward sweeps, respectively. The hysteresis-free mobility falls within this range.[10,11]

The threshold voltage V_T was extracted from a backward I_D–V_{GS} sweep at V_{DS} = 1 V by linear extrapolation.[12]

The conductivity mobility μ_{con} was calculated as $\mu_{con} = (R_{sh}\, p\, q)^{-1}$, where R_{sh} is the sheet resistance, $p = C_G\,(V_{GS} - V_T)$ is the hole density, and q is the elementary charge.

Table S1. Summary of monolayer WSe_2 microribbon FETs

Device	W [µm]	L [nm]	I_{max}[a)] [µA µm^{-1}]	μ_{FE} [cm^2 V^{-1} s^{-1}]	SS [V dec^{-1}]	V_T [V]
A	5.0	460	8.5	8	6.5	-60.8
B	5.0	3000	2.6	16	7.4	-60.9
C	5.0	270	8.7	4	6.8	-63.7
D	5.0	770	7.5	12	6.5	-60.6
E	5.0	970	5.9	12	7.7	-50.0
F	5.0	770	6.1	10	7.0	-56.8
G	5.0	470	8.2	8	6.3	-53.7

[a)]I_{max} was measured at V_{DS} = 1 V, with a carrier density ~ 5×10^{12} cm^{-2}

Table S2. Summary of passivated-edge monolayer WSe_2 nanoribbon FETs

Device	W [nm]	L [nm]	I_{max}[a)] [μA μm^{-1}]	μ_{FE} [cm^2 V^{-1} s^{-1}]	SS [V dec^{-1}]	V_T [V]
A	40	460	28±3	20±6	4.6±0.4	-52.2±1.4
B	50	3000	7±2	32±3	5.8±0.6	-57.5±1.7
C	56	270	57±2	18±5	5.8±0.3	-46.1±0.8
D	57	770	49±1	53±4	7.6±1.1	-33.8±0.5
E	60	970	46±3	47±4	4.9±0.5	-37.2±1.0
F	68	770	15±1	21±2	5.7±0.4	-50.0±1.0
G	70	470	20±4	34±8	4.4±0.4	-45.2±0.6

[a)]I_{max} was measured at V_{DS} = 1 V, with a carrier density ~ 5×10^{12} cm^{-2}

Table S3. Summary of open-edge monolayer WSe_2 nanoribbon FETs

Device	W [nm]	L [nm]	I_{max}[a] [µA µm^{-1}]	μ_{FE} [cm^2 V^{-1} s^{-1}]	SS [V dec^{-1}]	V_T [V]
A	40	460	1.0±0.4	1.4±0.5	8.7±1.0	-66.5±0.9
B	50	3000	0.042	2.0±0.7	16.4±0.5	-77.1±5.1
C	56	270	17.9±0.3	8.8±2.2	6.7±0.3	-59.8±0.5
D	57	770	2.9±0.5	5.4±0.5	10.2±0.6	-65.8±0.9
E	60	970	1.5±0.1	1.9±0.1	8.4±0.4	-49.5±3.3
F	68	770	1.2±0.2	2.7±0.3	11.3±1.3	-68.1±2.5
G	70	470	3.0±0.1	4.8±1.1	7.4±0.8	-55.9±0.5

[a] I_{max} was measured at V_{DS} = 1 V, with a carrier density ~ 5×10^{12} cm^{-2}

17. Cycle-to-cycle stability of passivated-edge nanoribbon FETs

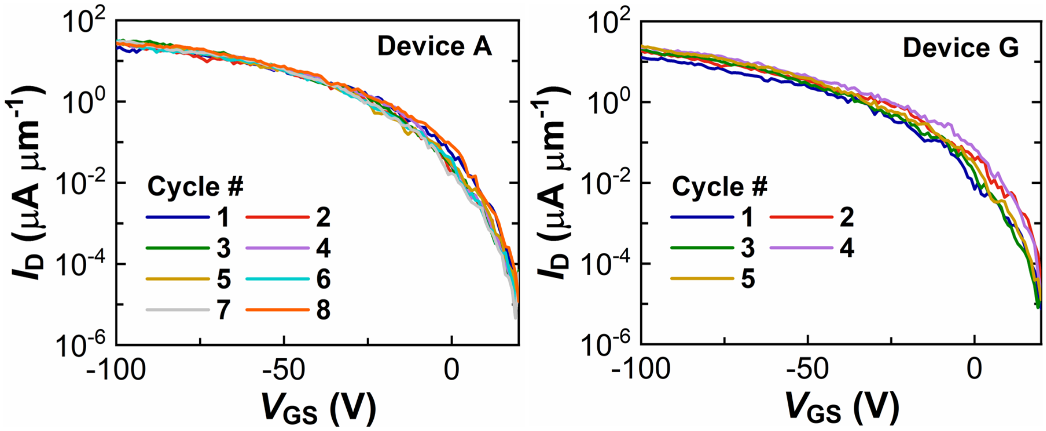

Figure S16. Transfer curves of two passivated-edge nanoribbon FETs (Devices A and G in Table S2) over multiple cycles at V_{DS} = 1 V. Error bars in this article include cycle-to-cycle variability.

18. Four-probe measurements

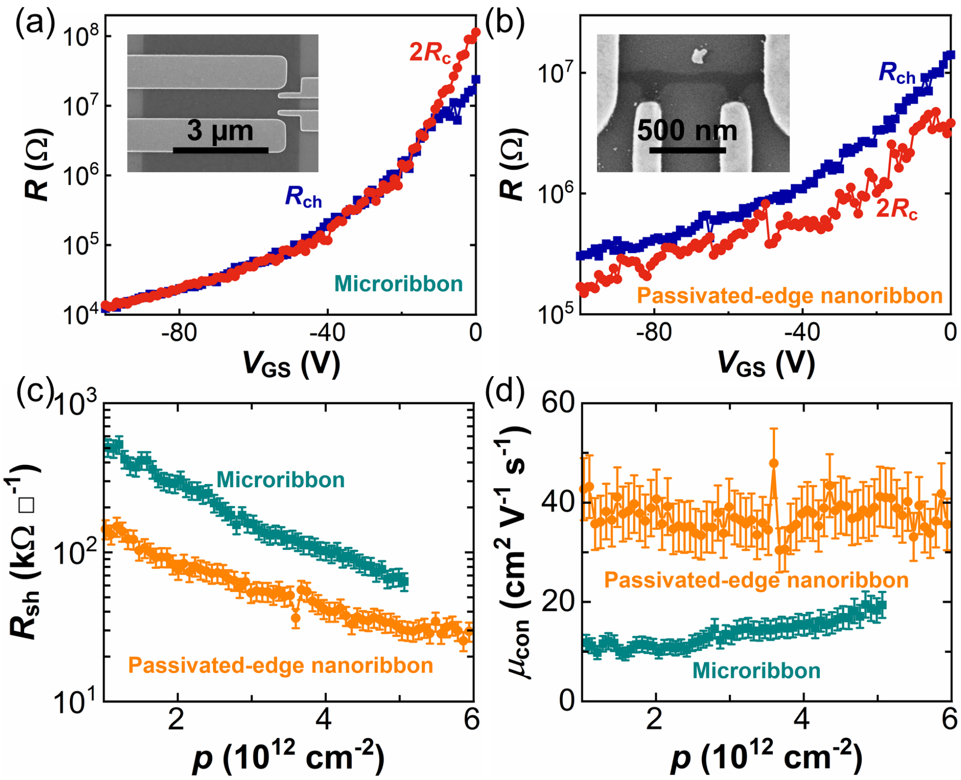

Figure S17. Four-probe measurements of a microribbon FET (W = 5 µm, L = 960 nm) and a passivated-edge nanoribbon FET (W = 55 nm, L = 960 nm). (a) Channel resistance R_{ch} and contact resistance $2R_c$ versus V_{GS} for the microribbon FET. The inset shows the SEM image of the microribbon FET. (b) Channel resistance R_{ch} and contact resistance $2R_c$ versus V_{GS} for the passivated-edge nanoribbon FET. The inset shows the SEM image of the nanoribbon FET with open edges. The $2R_c$ of the nanoribbon FET includes the junction resistances between the nanoribbon channel and the microribbon under the source/drain contacts, leading to a higher $2R_c$ in the nanoribbon FET compared to the microribbon FET. (c) Sheet resistance R_{sh} versus p. (d) Conductivity mobility μ_{con} versus p.

19. Additional sub-μm ribbon FETs for width dependence study

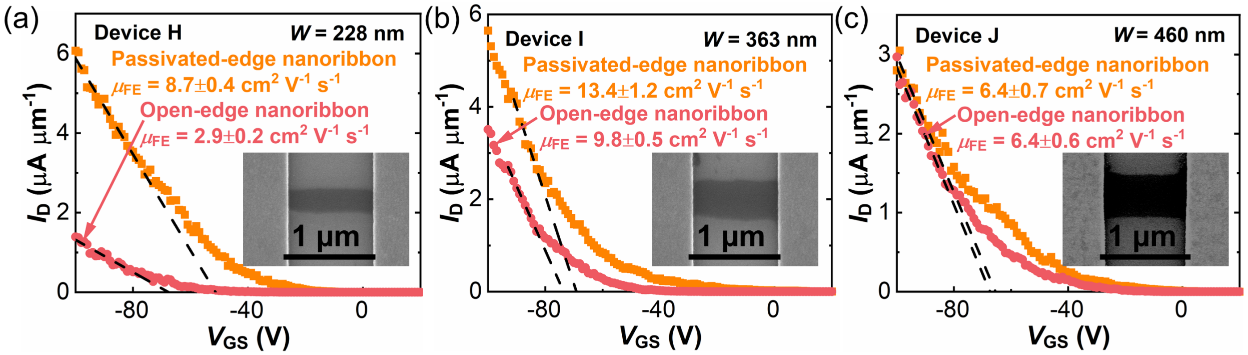

Figure S18. Transfer curves of monolayer WSe_2 FETs with varying channel widths in the range of 200–500 nm with passivated edges versus with open edges: (a) Device H (W = 228 nm, L = 930 nm). (b) Device I (W = 363 nm, L = 930 nm). (c) Device J (W = 460 nm, L = 940 nm). The insets show the SEM images of corresponding sub-μm ribbon FETs with open edges.

20. Dependence of I_{on} on W for nanoribbon FETs

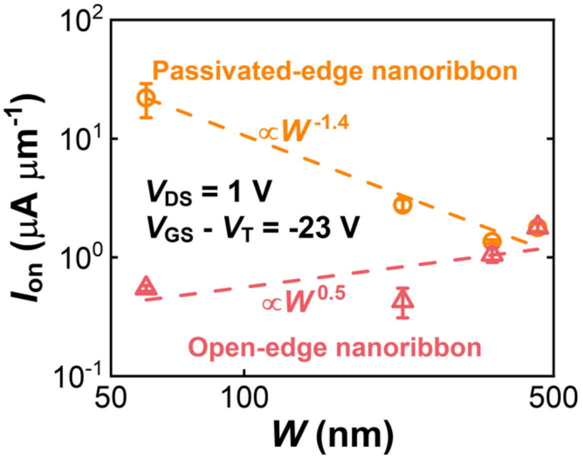

Figure S19. I_{on} versus W for both passivated-edge and open-edge nanoribbon FETs (Devices E, H–J) at $V_{DS} = 1$ V and $V_{GS} - V_T = -23$ V. The dashed lines are power-law fits to the data, showing I_{on} scales with W as $W^{-1.4}$ for passivated-edge nanoribbon FETs (Pearson's $r^2 = 0.987$), and $W^{0.5}$ for open-edge nanoribbon FETs ($r^2 = 0.46$).

21. Hysteresis analysis

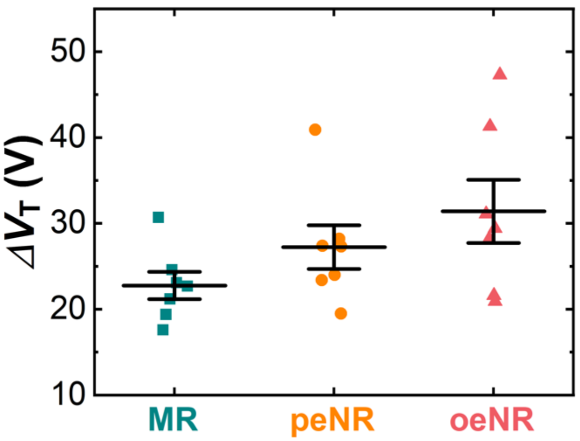

Figure S20. Hysteresis ΔV_T of Devices A–G as a microribbon (MR), passivated-edge nanoribbon (peNR), and an open-edge nanoribbon (oeNR), where ΔV_T is the difference between the threshold voltages V_T of backward and forward I_D–V_{GS} sweeps. Note that the surface of all nanoribbon FETs (both passivated-edge and open-edge) was passivated with a PMMA nanoribbon mask.

Table S4. Summary of reported TMD nanoribbon transistors

Polarity	TMD	$t^{\lozenge}$ [nm]	W [nm]	L [nm]	V_{DS} [V]	$I_{max}^{\lozenge\lozenge}$ [μA μm^{-1}]	μ_{FE} [cm^2 V^{-1} s^{-1}]	Fab method$^{\lozenge\lozenge\lozenge}$	Ref.
p	**WSe_2**	**1L**	**56**	**270**	**1**	**57**	**18**	**SPL + remote O_2 plasma**	**This work**
			**57**	**770**		**49**	**53**		
			**60**	**970**		**46**	**47**		
	WSe_2	4–12	240	300	1	0.0002	N.A.	SPL + H_2O etch	[13]
	WS_2	6L	50	40	1	2	3	CVD	[14]
	WSe_2	24	62	1000	1	0.003	N.A.	FIBIE	[15]
n	MoS_2	1L	30	160	1	2.5	9	SPL + XeF_2 etch	[1]
	MoS_2	1L	50	500	0.1	1	2	EBL + SF_6 plasma	[16]
	MoS_2	1L	50	500	1	29	50	EBL + SF_6 plasma	[17]
	MoS_2	1L	60	24	0.95	25	N.A.	EBL + etch	[18]
	MoS_2	1L	65–1080	N.A.	1	N.A.	10–35	CVD	[19]
	MoS_2	1L	125	1200	1	0.006	1.4	CVD	[20]
	MoS_2	1L	200	2600	1	0.002	0.12	SPL	[21]
	WS_2	1L	200	1000	1	0.28	0.4	CVD	[22]
	MoS_2	2L	8	400	0.1	8.4	4	APCVD	[23]
	MoS_2	5–15	65	50	1	23	17	EBL + SF_6 plasma	[24]
	MoS_2	6	40	1000	1	50	31	EBL + SF_6 plasma	[25]
	MoS_2	6	60	2000	1	10	N.A.	EBL + BCl_3 plasma	[26]
	MoS_2	7	140	200	0.5	20	36	CVT	[27]
	MoS_2	8	300	6300	1	0.001	N.A.	SPL + H_2O etch	[28]
	MoS_2	20	130	1000	1	14	53	NWL + Ar plasma	[29]
	MoS_2	>> 1L	170	3400	10	0.0006	0.003	laser writing	[30]

$^{\lozenge}$1L = monolayer, 2L = bilayer, etc

$^{\lozenge\lozenge}$The upper limit of the carrier density is set to 5×10^{12} cm^{-2} to ensure a fair comparison of I_{max} and I_{max}/I_{min}

$^{\lozenge\lozenge\lozenge}$SPL = scanning probe lithography, CVD = chemical vapor deposition, FIBIE = focused-ion-beam-induced etching, EBL = ebeam lithography, MOCVD = metal organic CVD, APCVD = atmospheric pressure CVD, CVT = chemical vapor transport, NWL = nanowire lithography

References

[1] S. Chen, S. Kim, W. Chen, J. Yuan, R. Bashir, J. Lou, A. M. van der Zande, W. P. King, *Nano Lett.* **2019**, *19*, 2092.

[2] S. Chen, W. P. King, *J. Vac. Sci. Technol. B* **2021**, *39*, 032601.

[3] A. Nipane, M. S. Choi, P. J. Sebastian, K. Yao, A. Borah, P. Deshmukh, Y. Jung, B. Kim, A. Rajendran, K. W. C. Kwock, A. Zangiabadi, V. M. Menon, P. J. Schuck, W. J. Yoo, J. Hone, J. T. Teherani, *ACS Appl. Mater. Interfaces* **2021**, *13*, 1930.

[4] G. Wei, E. J. Lenferink, D. A. Czaplewski, N. P. Stern, "Width-dependent Photoluminescence and Anisotropic Raman Spectroscopy from Monolayer MoS_2 Nanoribbons," can be found under http://arxiv.org/abs/1709.04001, **2017**.

[5] A. A. Shylau, J. W. Kłos, I. V. Zozoulenko, *Phys. Rev. B - Condens. Matter Mater. Phys.* **2009**, *80*, 205402.

[6] A. Behnam, A. S. Lyons, M. H. Bae, E. K. Chow, S. Islam, C. M. Neumann, E. Pop, *Nano Lett.* **2012**, *12*, 4424.

[7] J. Guo, Y. Yoon, Y. Ouyang, *Nano Lett.* **2007**, *7*, 1935.

[8] C. Lian, K. Tahy, T. Fang, G. Li, H. G. Xing, D. Jena, *Appl. Phys. Lett.* **2010**, *96*, 103109.

[9] R. K. A. Bennett, E. Pop, *Nano Lett.* **2023**, *23*, 1666.

[10] D. Estrada, S. Dutta, A. Liao, E. Pop, *Nanotechnology* **2010**, *21*, 085702.

[11] I. M. Datye, A. J. Gabourie, C. D. English, K. K. H. Smithe, C. J. McClellan, N. C. Wang, E. Pop, *2D Mater.* **2019**, *6*, 011004.

[12] D. K. Schroder, *Semiconductor Material and Device Characterization: Third Edition*, John Wiley & Sons, Hoboken, New Jersey, **2005**.

[13] A. I. Dago, Y. K. Ryu, R. Garcia, *Appl. Phys. Lett.* **2016**, *109*, 163103.

[14] C. C. Cheng, Y. Y. Chung, U. Y. Li, C. T. Lin, C. F. Li, J. H. Chen, T. Y. Lai, K. S. Li, J. M. Shieh, S. K. Su, H. L. Chiang, T. C. Chen, L. J. Li, H. S. P. Wong, C. H. Chien, in *Dig. Tech. Pap. - Symp. VLSI Technol.*, Institute Of Electrical And Electronics Engineers Inc., **2019**, pp. T244–T245.

[15] M. G. Stanford, P. R. Pudasaini, N. Cross, K. Mahady, A. N. Hoffman, D. G. Mandrus, G. Duscher, M. F. Chisholm, P. D. Rack, *Small Methods* **2017**, *1*, 1600060.

[16] D. Kotekar-Patil, J. Deng, S. L. Wong, K. E. J. Goh, *ACS Appl. Electron. Mater.* **2019**, *1*, 2202.

[17] D. Kotekar-Patil, J. Deng, S. L. Wong, C. S. Lau, K. E. J. Goh, *Appl. Phys. Lett.* **2019**, *114*, 13508.

[18] K. P. O'Brien, C. J. Dorow, A. Penumatcha, K. Maxey, S. Lee, C. H. Naylor, A. Hsiao, B. Holybee, C. Rogan, D. Adams, T. Tronic, S. Ma, A. Oni, A. Sen Gupta, R. Bristol, S. Clendenning, M. Metz, U. Avci, in *Tech. Dig. - Int. Electron Devices Meet. IEDM*, Institute Of Electrical And Electronics Engineers Inc., **2021**, pp. 7.1.1-7.1.4.

[19] S. Li, Y. C. Lin, W. Zhao, J. Wu, Z. Wang, Z. Hu, Y. Shen, D. M. Tang, J. Wang, Q. Zhang, H. Zhu, L. Chu, W. Zhao, C. Liu, Z. Sun, T. Taniguchi, M. Osada, W. Chen, Q. H. Xu, A. T. S. Wee, K. Suenaga, F. Ding, G. Eda, *Nat. Mater.* **2018**, *17*, 535.

[20] C. Yang, B. Wang, Y. Xie, Y. Zheng, C. Jin, *Nanotechnology* **2019**, *30*, 255602.

[21] F. M. Espinosa, Y. K. Ryu, K. Marinov, D. Dumcenco, A. Kis, R. Garcia, *Appl. Phys. Lett.* **2015**, *106*, 103503.

[22] H. Suzuki, M. Kishibuchi, M. Misawa, K. Shimogami, S. Ochiai, T. Kokura, Y. Liu, R. Hashimoto, Z. Liu, K. Tsuruta, Y. Miyata, Y. Hayashi, *ACS Nano* **2023**, *17*, 9455.

[23] X. Li, B. Li, J. Lei, K. V. Bets, X. Sang, E. Okogbue, Y. Liu, R. R. Unocic, B. I. Yakobson, J. Hone, A. R. Harutyunyan, *Sci. Adv.* **2021**, *7*, 1892.

[24] B. Stampfer, F. Zhang, Y. Y. Illarionov, T. Knobloch, P. Wu, M. Waltl, A. Grill, J. Appenzeller, T. Grasser, *ACS Nano* **2018**, *12*, 5368.

[25] F. Zhang, C.-H. Lee, J. A. Robinson, J. Appenzeller, *Nano Res.* **2018**, *11*, 1768.

[26] H. Liu, J. Gu, P. D. Ye, *IEEE Electron Device Lett.* **2012**, *33*, 1273.

[27] P. Paletti, S. Fathipour, M. Remškar, A. Seabaugh, *J. Appl. Phys.* **2020**, *127*, 65705.

[28] Y. K. Ryu, A. I. Dago, Y. He, F. M. Espinosa, E. López-Elvira, C. Munuera, R. Garcia, *Appl. Surf. Sci.* **2021**, *539*, 148231.

[29] X. Duan, Z. Yang, J. Lin, H. Huang, G. Li, D. Wan, X. Zou, J. Bai, J. Miao, L. Liao, X. Liu, *IEEE Trans. Electron Devices* **2022**, *69*, 3433.

[30] P. Zuo, L. Jiang, X. Li, M. Tian, C. Xu, Y. Yuan, P. Ran, B. Li, Y. Lu, *ACS Appl. Mater. Interfaces* **2019**, *11*, 39334.
